# *Shank3* Mice Carrying the Human Q321R Mutation Display Enhanced Self-Grooming, Abnormal Electroencephalogram Patterns, and Suppressed Neuronal Excitability and Seizure Susceptibility

**DOI:** 10.3389/fnmol.2019.00155

**Published:** 2019-06-18

**Authors:** Ye-Eun Yoo, Taesun Yoo, Seungjoon Lee, Jiseok Lee, Doyoun Kim, Hye-Min Han, Yong-Chul Bae, Eunjoon Kim

**Affiliations:** ^1^Department of Biological Sciences, Korea Advanced Institute of Science and Technology, Daejeon, South Korea; ^2^Center for Synaptic Brain Dysfunctions, Institute for Basic Science, Daejeon, South Korea; ^3^Department of Anatomy and Neurobiology, School of Dentistry, Kyungpook National University, Daegu, South Korea

**Keywords:** Shank3, autism spectrum disorder, patient mutations, self-grooming, anxiety-like behavior, excitability, EEG, seizure susceptibility

## Abstract

Shank3, a postsynaptic scaffolding protein involved in regulating excitatory synapse assembly and function, has been implicated in several brain disorders, including autism spectrum disorders (ASD), Phelan-McDermid syndrome, schizophrenia, intellectual disability, and mania. Here we generated and characterized a *Shank3* knock-in mouse line carrying the Q321R mutation (*Shank3*^Q321R^ mice) identified in a human individual with ASD that affects the ankyrin repeat region (ARR) domain of the Shank3 protein. Homozygous *Shank3*^Q321R/Q321R^ mice show a selective decrease in the level of Shank3a, an ARR-containing protein variant, but not other variants. CA1 pyramidal neurons in the *Shank3*^Q321R/Q321R^ hippocampus show decreased neuronal excitability but normal excitatory and inhibitory synaptic transmission. Behaviorally, *Shank3*^Q321R/Q321R^ mice show moderately enhanced self-grooming and anxiolytic-like behavior, but normal locomotion, social interaction, and object recognition and contextual fear memory. In addition, these mice show abnormal electroencephalogram (EEG) patterns and decreased susceptibility to induced seizures. These results indicate that the Q321R mutation alters Shank3 protein stability, neuronal excitability, repetitive and anxiety-like behavior, EEG patterns, and seizure susceptibility in mice.

## Introduction

Shank proteins, a family of postsynaptic scaffolding proteins with three known members, have been implicated in the regulation of excitatory synapse assembly and function (Sheng and Kim, 2000, 2011; Sheng and Sala, 2001; Boeckers et al., 2002; Sheng and Hoogenraad, 2007; Grabrucker et al., 2011; Jiang and Ehlers, 2013; Sala et al., 2015; Monteiro and Feng, 2017; Mossa et al., 2017). The third member of the family, Shank3, also known as ProSAP2, contains multiple domains for protein-protein interactions, including an SPN (Shank/ ProSAP N-terminal) domain, ankyrin repeats, an SH3 domain, a PDZ domain, a proline-rich region, and a SAM (sterile alpha motif) domain (Du et al., 1998; Boeckers et al., 1999; Lim et al., 1999; Naisbitt et al., 1999; Sheng and Kim, 2000). These domains mediate interactions with diverse synaptic proteins, including GKAP (also known as SAPAP1 and DLGAP1) and Homer.

Clinically, *SHANK3* has been implicated in multiple neurodevelopmental and psychiatric disorders, including autism spectrum disorders (ASD), Phelan-McDermid syndrome (PMS), schizophrenia, intellectual disability, and mania (Bonaglia et al., 2001; Wilson et al., 2003; Durand et al., 2007; Moessner et al., 2007; Gauthier et al., 2010; Bonaglia et al., 2011; Hamdan et al., 2011; Leblond et al., 2012; Boccuto et al., 2013; Han et al., 2013; Guilmatre et al., 2014; Leblond et al., 2014; Cochoy et al., 2015; Nemirovsky et al., 2015; de Sena Cortabitarte et al., 2017; De Rubeis et al., 2018). Importantly, *SHANK3* mutations have been shown to account for ∼1% of all ASD cases (Leblond et al., 2014). Multiple lines of *Shank3*-mutant mice and, more recently, rats that carry global, conditional and point mutations in *Shank3*, have been generated and characterized, providing information about normal and disease-related functions of Shank3 (Bozdagi et al., 2010; Peca et al., 2011; Wang et al., 2011; Schmeisser et al., 2012; Yang et al., 2012; Han et al., 2013; Kouser et al., 2013; Lee et al., 2015; Speed et al., 2015; Jaramillo et al., 2016, 2017; Mei et al., 2016; Wang et al., 2016; Zhou et al., 2016; Harony-Nicolas et al., 2017; Vicidomini et al., 2017; Amal et al., 2018; Berg et al., 2018; Bey et al., 2018; Drapeau et al., 2018; Engineer et al., 2018; Fourie et al., 2018; Heise et al., 2018; Jin et al., 2018; Ma et al., 2018; Qin et al., 2018; Yoo et al., 2018; Zhu et al., 2018; Balaan et al., 2019; Rendall et al., 2019). These animals display diverse synaptic, neuronal, circuit and behavioral abnormalities, providing substantial insight into how *Shank3* mutations lead to various phenotypic abnormalities in mice (Jiang and Ehlers, 2013; Harony-Nicolas et al., 2015; Sala et al., 2015; Ferhat et al., 2017; Monteiro and Feng, 2017; Mossa et al., 2017; Tan and Zoghbi, 2018). However, with the exception of recent studies on two mouse lines carrying an ASD-linked InsG3680 mutation and a schizophrenia-linked R1117X mutation (Zhou et al., 2016) and a mouse line carrying the S685I mutation (Wang et al., 2019), mouse lines expressing point mutations of *Shank3* identified in human individuals with ASD, PMS, or other disorders have not been reported.

The Shank3 Q321R mutation was identified as a de novo mutation in an individual with ASD who displayed symptoms including social and language deficits, repetitive behaviors (verbal repetitive behaviors, hair pulling, but no motor stereotypies), restricted interests, inattention and irritability (Moessner et al., 2007). This mutation has been shown to decrease excitatory synaptic targeting of Shank3 and Shank3-dependent dendritic spine development, decrease F-actin levels in spines, and suppress excitatory synaptic transmission in cultured hippocampal neurons (Durand et al., 2012). In a more recent study, this mutation was shown to enhance the interaction of Shank3 with Sharpin, but not with α-fodrin (Mameza et al., 2013), two known ligands of the ARR (ankyrin repeat region) domain of Shank3 (Bockers et al., 2001; Lim et al., 2001). In addition, the Q321R mutation has stronger influences on excitatory synapses, as compared with other Shank3 mutations such as R12C and R300C (Durand et al., 2012). These results indicate that the Q321R mutation exerts a significant influence on ASD-related behaviors and excitatory synapse development and function. However, *in vivo* functions of the Q321R mutation have not been explored.

In the present study, we generated and characterized a new *Shank3*-mutant mouse line carrying the Q321R mutation (*Shank3*^Q321R^ mice) and studied its *in vivo* effects. We found that this mutation leads to destabilization of Shank3 protein, decreased excitability in hippocampal CA1 pyramidal neurons, enhanced self-grooming and anxiolytic-like behavior, altered electroencephalogram (EEG) patterns, and decreased seizure susceptibility.

## Materials and Methods

### Structural Modeling of the Shank3 Protein Containing a Q321R Mutation

The structure of the SPN and ARR domains of the mouse Shank3 protein containing the p.Q321R missense mutation was modeled using the mutagenesis function in PyMOL software (version 1.3) (DeLano, 2009) based on the crystal structure of the SPN and ARR domains of the rat Shank3 protein (PDB ID: 5G4X). Energy minimization and loop flexible modeling were performed using Modeller software (Fiser et al., 2000). Electrostatic charge distribution surfaces were calculated and represented using PyMOL software (version 1.3) (DeLano, 2009). All structural figures were prepared using PyMOL software (version 1.3) (DeLano, 2009).

### Stability Prediction of Mutant Shank3 Proteins

The stability of the SPN and ARR domains of Shank3 containing the ASD-risk missense mutations, p.R12C, p.L68P, p.A198G, p.R300C, or p.Q321R, were predicted using the *in silico* algorithm in I-Mutant 2.0 (version 2.0)^1^ under conditions of pH 7.0 and 25°C (Capriotti et al., 2005). I-Mutant 2.0 is a support vector machine (SVM)-based web server for automatic prediction of stability changes upon a single point mutation. We used the crystal structure of the SPN and ARR domains of Shank3 protein (PDB ID: 5G4X) as a template structure to calculate changes in the stability of the protein containing ASD-risk missense mutations. The ΔΔG value was calculated by subtracting the unfolding Gibbs free energy of wild-type (WT) protein from that of the mutated protein (kcal/mol). A negative ΔΔG value indicates a decrease in the stability of the mutated protein.

### Animals

A mouse embryonic stem (ES) cell line harboring the Shank3 Q321R mutation was generated by Cyagen. ES cells were injected into C57BL/6N blastocysts to produce chimeric mice, which were crossed with WT C57BL/6J to produce heterozygous knock-in (KI) F1 mice. F1 mice were then crossed with *protamine-Cre* mice to remove the Neo cassette, followed by backcrossing into a C57BL/6J background for more than five generations. All mice used for experiments in the present study were obtained by mating heterozygotes (HT x HT). The average WT:HT:KI ratio was 0.94:1.94:1.12, consistent with the expected 1:2:1 Mendelian ratio. Breeding was successful in >95% of cases, and homozygous *Shank3*^Q321R^ mice showed survival rates and body weights comparable to those of WT mice. The mice were fed *ad libitum* under 12-h light-dark cycles, and 2–6 mice were grouped in each cage. Mice were bred and maintained according to the Animal Research Requirements of KAIST, and all procedures were approved by the Committee of Animal Research at KAIST (2016-30). For PCR genotyping, the following oligonucleotide primers were used to detect a band of 478 base pairs for WT allele and a band of 622 base pairs for the Q321R mutant allele: forward, 5′-CAT GAG GCA CCC TTT TCT GT-3′, reverse, 5′-TGT CCC TAA CCC CAA TGT GT-3′.

### Western Blot

Brains from *Shank3*^Q321R^ mice (*Shank3*^Q321R/Q321R^ and *Shank3*^+/Q321R^) and their WT littermates (3 months) were extracted in ice-cold homogenization buffer (0.32 M sucrose, 10 mM HEPES, 2 mM EDTA, 2 mM EGTA, protease inhibitors and phosphatase inhibitors) and homogenized by motorized tissue grinder. Brain whole lysates were prepared by boiling with β-mercaptoethanol. After immunoblotting with antibodies to Shank3 (Santa Cruz SC-30193; H-160) and α-tubulin (Sigma T5168), fluorescent secondary antibody signals were detected using Odyssey Fc Dual Mode Imaging System.

### Immunohistochemistry

Adult mice (2 months, female) were anesthetized and perfused transcardially with 4% paraformaldehyde. Brains were removed and post-fixed overnight at 4°C followed by Vibratome sectioning (50 μm; Leica VT 1200S). After washing in phosphate-buffered saline (PBS), brain sections were blocked in 5% bovine serum albumin (BSA) in PBS for 3–5 h. Brain sections were incubated with primary antibodies (NeuN, 1:1000, Millipore) in 5% BSA overnight at 4°C and washed three times for 20 min in 0.2% TritonX-100 in PBS (PBST). After incubation with Alexa 594 secondary antibody (1:500, Jackson ImmunoResearch) at room temperature for 1 h, sections were washed three times for 10 min in PBST. Brain sections were mounted with DAPI mounting medium. Brain sections were imaged with a confocal microscope (10x objective; LSM780, Carl Zeiss).

### Behavioral Assays

Behavioral analyses were performed during light-off periods on male 2–4-month-old male or female mice in their home cages. WT and mutant littermates (partly pairs) from several mother mice were used to form a cohort. All mice were handled for 10 min per day for 3 days by experienced researchers prior to behavioral experiments. Mouse behavioral assays were performed in the following order: Laboras monitoring of 96-h movements, open-field test, novel-object–recognition test, repetitive behaviors, three-chamber social-interaction test, elevated plus-maze (EPM) test, light-dark test, courtship adult ultrasonic vocalization (USV), and contextual fear conditioning.

#### Laboras

Long-term locomotion and behaviors of mice were recorded and analyzed using the Laboratory Animal Behavior Observation Registration and Analysis System (LABORAS^TM^) by Metris. Mice without habitation to the behavioral booth or Laboras cages were put into Laboras cages where recordings were conducted for 96 consecutive hours. Laboras results were not validated by manual analyses because similar Laboras validations have been reported (Van de Weerd et al., 2001; Quinn et al., 2003; Quinn et al., 2006; Dere et al., 2015).

#### Open-Field Test

The open-field test was performed as described previously (Gould et al., 2009). A subject mouse was gently introduced into the center zone of the white acryl chamber (40 × 40 × 40 cm) and recorded of movements for 1 h. Locomotor activities and time spent in the center region (20 × 20 cm) of the open field arena were analyzed using EthoVision XT 10 (Noldus).

#### Novel Object Recognition Test

Novel object recognition memory (Antunes and Biala, 2012) was measured as follows. Briefly, after 1-h habituation in the white acryl chamber 1 day before the experimental day, the subject mouse was allowed to explore two objects of the same shape and material placed symmetrically in the center of the chamber for 10 min. Twenty-four hour later, one object was replaced by a novel object, and the subject mouse was allowed to explore the two objects (one novel and the other familiar) for 10 min. Exploration was defined as events in which the mouse sniffs the object within 1 cm distance or climbs up the object. Time spent in sniffing each object was analyzed by EthoVision XT 10 (Noldus) using the movies from the first 5 min to minimize saturation effects. Genotype comparisons were made using the discrimination index [time spent exploring novel object – time spent exploring familiar object)/(time spent exploring novel + familiar objects) ^∗^ 100], as previously described (Stefanko et al., 2009; Vogel-Ciernia and Wood, 2014).

#### Repetitive Behaviors

Autism-relevant repetitive behaviors (Silverman et al., 2010) was measured as follows. The subject mice were allowed to freely move in a clean new home cage with bedding (∼60 lux). Time spent in digging and self-grooming during the 10-min session from a single recording was measured manually in a blind manner. Digging was defined as a mouse uses its head or forelimbs to dig out beddings. Self-grooming was defined as a mouse stroking or scratching its face or body area, or licking its body.

#### Three-Chamber Social Interaction Test

The three-chamber test, known to measure social approach and social novelty-recognition behavior (Moy et al., 2004; Nadler et al., 2004; Silverman et al., 2010), was performed as previously described (Lee et al., 2015). Briefly, the subject mice were isolated for 4 days before this assay. Three-chambered apparatus (60 × 40 × 20 cm) consisted of the left, center, and right chamber with two entrances to the center chamber. Two empty containers were located in the corner of left and right chamber but not the center chamber. This assay consisted of three sessions. First, the subjects were allowed to freely explore all three chambers for 10 min. Second, the subject mouse was allowed to explore the containers with a stranger 1 (S1) or a novel object (O) for 10 min. Finally, the novel object was replaced with a stranger 2 (S2), and the subject mouse was allowed to explore S1 and S2 for 10 min. During the interval between each session, the subject was gently guided to the center chamber and the two entrances were blocked. Analysis of time spent in sniffing stranger/object was performed using EthoVision XT 10 (Noldus)

#### Elevated Plus-Maze Test

The EPM test (Pellow et al., 1985) was performed as follows. Briefly, the EPM apparatus, located 75 cm above the floor, consisted of two open arms (30 × 5 × 0.5 cm), two closed arms (30 × 5 × 30 cm), and a center zone with access to both arms. Light conditions were 180 lux for open arms and 20 lux for closed arms. A subject mouse was gently introduced into the center zone and allowed to move freely the open and closed arms for 8 min. Time spent in open/closed arms was measured using EthoVision XT 10 (Noldus).

#### Light-Dark Test

The light-dark test (Bourin and Hascoet, 2003) was performed as previously described (Ikeda et al., 1995) with minor modifications. Animals were placed in the light chamber with their heads toward the opposite wall from the dark chamber, and allowed to explore the light-dark apparatus (20 × 30 × 20 cm, 670 lux for light chamber, 20 × 13 × 20 cm, 5 lux for dark chamber), which has a 5-cm wide entrance between the two chambers. Time spent in the light chamber was analyzed using EthoVision XT 10 (Noldus).

#### Direct Social Interaction Test

Direct social interaction test was performed as described previously (Chung et al., 2015). Mice, which were isolated in their home cage for 4 days, were used in this assay. On the day of habituation, mice were individually habituated in gray acryl box (33 × 33 × 22 cm) for 10 min. On the day for experiments, two mice in the same genotype that never met each other previously were put into the habituated box and allowed to freely interact each other for 10 min while all behaviors were recorded. Time spent in interaction such as nose-to-nose sniffing, following, nose-to-tail sniffing, and other interactions such as body contacts and huddling were measured manually in a blind manner.

#### Courtship Adult Ultrasonic Vocalization

Courtship ultrasonic vocalizations (Portfors, 2007) was measured as follows. Male adult subject mice were socially isolated in their home cages for 4 days to allow them to recognize the cage as their own territory. Female adult stranger mice were group-caged prior to these experiments on the assumption that group housing might synchronize female cycles. A subject male mouse was habituated to a novel test cage for 5 min while recording its basal vocalizations in the absence of a female stranger. Next, a female stranger mouse was introduced into the subject’s cage, and the two mice were allowed to interact freely with each other while recording courtship USVs of the subject mouse for 5 min. Because courtship USVs in the context of male-female encounters are mainly produced by males (Maggio and Whitney, 1985; Egnor and Seagraves, 2016), USVs were not categorized as arising from male or female mice. Avisoft SASLab Pro was used to analyze USVs.

#### Contextual Fear Conditioning and Extinction Test

Contextual fear conditioning and extinction (Phillips and LeDoux, 1992; Milad and Quirk, 2002) were measured as described previously (Lee et al., 2012). The fear conditioning test consisted of two sessions performed on consecutive days. On day 1, subject mice were introduced to the conditioning box and allowed to freely explore the environment for 120 s, and then received a series of 0.8 mA electric foot shocks (1 s; without associated auditory cue) for five times at intervals of 120 s. After the last shock the mice were left in the box for an additional 120 s, making the total experimental time 12 min. On day 2, the mice were placed in the same conditioning box and allowed to freely explore for 10 min without any stimuli, and the freezing levels of the mice on the test day (day 2) were quantified for the first 3 min. These mice, which were tested for 24-h fear memory, were exposed to the same context for 5 min to measure fear extinction every day during the following 7 days (days 3–9). All freezing behaviors were recorded and analyzed using FreezeFrame software (Coulbourn Instruments).

#### Hot Plate Test

Hot plate test was performed as previously described (Ankier, 1974) with minor modifications. The subject mice were placed in the clear acryl chamber (10 × 10 × 15 cm), which was located on an aluminum heat controller (Sun Electronics Co.) maintained at 50–51°C. Times taken for the animals to lick their fore-/hindpaws or to jump were measured.

#### Von Frey Test

The von Frey test was performed according to the standard operating procedures of the Jackson Laboratory Mouse Neurobehavioral Phenotyping Facility (JAX-MNBF). The von Frey test apparatus consisted of a wire floor grid and a clear observation arena with aerated lids (Stoelting Co.). A subject mouse was gently introduced in the observation arena and habituated for 1 h until the activity levels become low. Beginning with the left hind paw, stimuli of von Frey filaments (Stoelting Co.) were presented perpendicularly to the plantar surface of the hind paws of the subject mice for two trials. The initial stimulus started with a von Frey filament with 0.4 g bending force. When there is a withdrawal response, the strength of the stimulus was gradually decreased to lower bending forces (0.16 to 0.02 g), while the stimulus was increased to a higher bending force (0.6 g) when there was no response. The minimum threshold required to induce a withdrawal response for left and right paws was recorded. A withdrawal response is defined as the mouse lifting up their hind paws when a filament is pushed with increasing pressure until it bends. All experimental procedures and analysis were performed in a double-blinded manner.

### Electrophysiology

Male or female mice were used for electrophysiological measurements (details are described in figure legends). After anesthetization with isoflurane, mouse brains were removed, and sagittal sections (300 μm) including hippocampus or striatum were prepared using a Vibratome (Leica VT 1200S) in ice-cold section buffer (in mM: 212 sucrose, 25 NaHCO_3_, 5 KCl, 1.25 NaH_2_PO_4_, 10 D-glucose, 1.2 L-ascorbic acid, 2 Na-pyruvate, 3.5 MgSO_4_, 0.5 CaCl_2_). Slices were maintained in artificial cerebrospinal fluid (ACSF) (in mM: 124 NaCl, 25 NaHCO_3_, 10 glucose, 2.5 KCl, 1 NaH_2_PO_4_, 2.5 CaCl_2_, 1.25 MgCl_2_) bubbled with 95% O_2_ and 5% CO_2_ for 30 min at 32°C followed by incubation for 30 min at room temperature. For mEPSC measurements, ACSF contained tetrodotoxin (0.5 μM) and picrotoxin (60 μM). Whole-cell recordings of pyramidal neurons in the hippocampal CA1 region were performed using a recording pipette filled with internal solution (in mM: 100 CsMeSO_4_, 10 TEA-Cl, 8 NaCl, 10 HEPES, 5 QX-314-Cl, 2 Mg-ATP, 0.3 Na-GTP and 10 EGTA with pH 7.25, 295 mOsm). For mIPSC measurement, ACSF contained tetrodotoxin (0.5 μM), NBQX (10 μM), and AP5 (50 μM). Whole-cell recordings of pyramidal neurons in the hippocampal CA1 region were performed with a recording pipette filled with internal solution (in mM: 115 CsCl, 10 TEA-Cl, 8 NaCl, 10 HEPES, 5 Qx-314-Cl, 4 Mg-ATP, 0.3 Na-GTP, 10 EGTA with pH 7.35, 295 mOsm). For neuronal excitability measurement, ACSF contained picrotoxin (60 μM), NBQX (10 μM), and AP5 (50 μM). Whole-cell recordings in pyramidal neurons in the hippocampus CA1 region were performed with a recording pipette filled with internal solution (in mM: 137 K-gluconate, 5 KCl, 10 HEPES, 0.2 EGTA, 10 Na_2_-phosphocreatine, 4 Mg-ATP, 0.5 Na-GTP with pH 7.2, 280 mOsm). First minimal currents were introduced to hold the membrane potential around −70 to −75 mV in a current clamp mode. To evoke depolarizing voltage sag responses, increasing amounts of depolarizing step currents (by 10 pA, −150 to 20 pA) were injected. Then, to elicit action potentials, increasing amounts of depolarizing currents (0 to 330 pA) were injected in a stepwise manner. Input resistance was calculated as the linear slope of current-voltage plots generated from a series of increasing current injection steps. The sag ratio was determined as the ratio of a steady state value to repolarized peak at the entire length of injected current at each step. The synaptic responses were amplified (Multiclamp 700B, Molecular Devices) and digitized (Digidata 1550, Molecular Devices) for analyses. mE/IPSCs were analyzed using Clamfit 10.4 software and horizontally spread-out currents where the high noise levels that are visible in horizontally compressed currents do not inhibit accurate analyses of the currents.

### Electroencephalography

Electroencephalography (EEG) recording was performed as previously described with minor modifications (Lee et al., 2018). An EEG driver was composed of a small header pin connector socket (Hirose Electric, H2021-ND) connected with six stainless steel screws (1 mm × 3 mm) through soldered coated stainless steel wire. All screws were implanted on the skull (two for bilateral frontal, +1.8 mm AP, ±1.0 mm ML; two for bilateral parietal, −2.0 mm AP and ±1.8 mm ML; two for animal ground and reference, −1.0 mm AP and ±1.0 mm ML from lambda). After recovery 1 week after the surgery, EEG recordings were performed using a Cheetah Data Acquisition System (Neuralynx). The subject mice were allowed to freely move around in a white acryl box (25 × 25 × 35 cm) with synchronized video recording. After 20-min habituation in the box, EEGs were recorded for 40 min. EEG data were analyzed using a customized MATLAB code. Baseline EEGs were analyzed using 5-min serial samplings, and the total power spectral density (PSD) was averaged per mouse by applying Fast Fourier Transform (FFT). EEG frequency ranges were defined as follows; Delta, 0–4 Hz; theta, 4–12 Hz; alpha, 12–30 Hz; low gamma, 30–80 Hz; high gamma, 80–130 Hz.

### PTZ-Induced Seizure Susceptibility

To determine the susceptibility of mice to PTZ-induced seizures, we performed the experiments using mice that did not receive EEG driver surgery for cleaner results. After intraperitoneal injection of PTZ (Sigma; 50 mg/kg), subject mice were placed in a clean new home cage. Video recordings for 20 min were used to analyze seizure stages defined as follows; stage 1, behavioral arrest; stage 2, myoclonic (jerk) seizures; stage 3: general tonic-clonic seizures, as previously described (Naydenov et al., 2014). The seizure susceptibility score was defined as follows; 0.2 × (latency to stage 1) + 0.3 × (latency to stage 2) + 0.5 × (latency to stage 3).

### Statistical Analysis

All behavioral, biochemical, and electrophysiological experiments were performed by researchers blinded to the experimental conditions. Statistical analyses were performed using GraphPad Prism 7 and MATLAB. Data sets, after removal of outliers, were analyzed for significance using unpaired two-tailed Student’s *t*-test, Mann–Whitney *U*-test, one-sample *t*-test, log-rank test, and two-way analysis of variance (ANOVA) with *post hoc* multiple comparisons test when appropriate. The n values and statistical significance values are indicated in figure legends. Statistical significance values are indicated as follows in the figures: ^∗^*P* < 0.05; ^∗∗^*P* < 0.01; ^∗∗∗^*P* < 0.001; ns, not significant. Additional statistical details and results, as well as information on sex, age and numbers of mice used, are presented in Supplementary Table S1.

## Results

### Molecular Modeling of the Shank3 Q321R Mutation

The Q321R mutation in the *SHANK3* gene (Moessner et al., 2007), which strongly influences the synaptic targeting of Shank3 and the development and function of dendritic spines and excitatory synapses (Durand et al., 2012), is located in the ARR domain of the protein. The ARR domain of Shank3 has been shown to interact with the N-terminal SPN domain of Shank3 in an intramolecular manner, leading to suppression of the binding of the ARR domain to Sharpin and α-fodrin (Mameza et al., 2013). These results suggest the possibility that these intra- and intermolecular interactions of the SPN and ARR domains of Shank3 might underlie the effects of the Q321R mutation.

**FIGURE 1:**
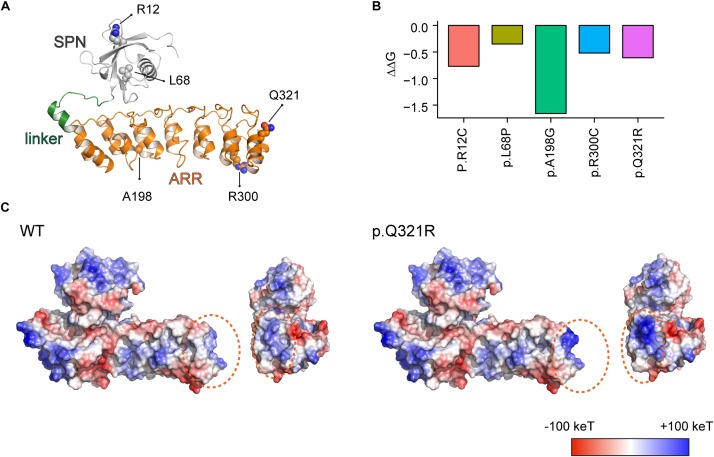
Molecular modeling-based predictions of protein stability and surface charge distribution in SPN-ARR domains of the Shank3 protein harboring Q321R and other ASD-risk mutations. **(A)** Structure of the SPN and ARR domains of the Shank3 protein (PDB ID: 5G4X), with the locations of amino acid residues mutated in autistic individuals (R12C, L68P, A198G, R300C, and Q321R) indicated. The SPN domain, the linker connecting the SPN and ARR domains, and the ARR domain of Shank3 are indicated in gray, green, and orange colors, respectively, as a ribbon diagram. The ASD-risk residues are indicated by ball-and-stick models. **(B)** Stability predictions of SPN and ARR domains of the Shank3 protein containing the indicated ASD-risk mutations, obtained using I-Mutant 2.0 software. A negative predicted free energy change (ΔΔG, in kcal/mol) indicates a decrease in the stability of the mutant protein. **(C)** Electrostatic surface charge distribution patterns in WT and mutant (Q321R) SPN and ARR domains of the Shank3 protein. Negative and positive surface charges are indicated in red and blue, respectively. Hydrophobic surfaces are indicated in white. Surface areas with the Shank3 Q321R mutation are indicated by dotted orange circles.

Using the recently reported X-ray crystal structure of the N-terminal SPN and ARR domains of Shank3 (Lilja et al., 2017), we attempted molecular modeling of the SPN and ARR domains harboring the Q321R mutation to determine whether the mutation could interfere with the interaction between these two domains (Figure 1A). We also included the following additional ASD-risk Shank3 mutations in SPN and ARR domains in our modeling (Durand et al., 2007; Moessner et al., 2007; Gauthier et al., 2010): L68P, which disrupts the interaction between the SPN and ARR domains and enhances the interactions of the ARR with Sharpin (Mameza et al., 2013); R12C, which affects excitatory synapse structure and function (Durand et al., 2012) and disrupts SPN binding to GTP-bound Ras and Rap small GTPases to suppress integrin signaling (Lilja et al., 2017); and R300C, which affects excitatory synapse structure and function (Durand et al., 2012).

We found that the Q321R mutation, unlike the L68P mutation, is located in the C-terminal region of the ARR domain, away from the interface of the SPN and ARR domains, and thus is unlikely to affect the SPN-ARR interaction (Figure 1A). We next tested whether these mutations affect the stability of SPN and ARR domains by calculating changes in free energy. All five mutations, including the Shank3 Q321R mutation, induced significant decreases in the stability of the protein, with the A198G mutation exerting the strongest effect (Figure 1B), although the impact of the A198G mutation has not been explored in previous *in vitro* studies (Durand et al., 2012; Mameza et al., 2013). In addition, an analysis of surface charge distribution indicated that the Q321R mutation induces a substantial increase in the local positive charge density (Figure 1C). These results suggest that the Q321R mutation is less likely to affect the intramolecular SPN-ARR interaction, but more likely to affect the protein stability or intermolecular interactions of Shank3.

### Generation and Characterization of *Shank3*^Q321R^ Mice

**FIGURE 2:**
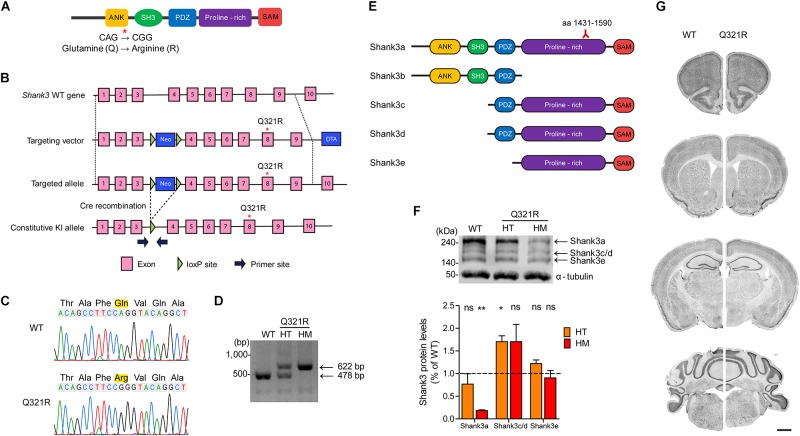
Generation and characterization of *Shank3*^Q321R^ mice. **(A)** A schematic diagram showing the location of the Shank3 Q321R mutation in the Shank3 protein. Ank, ankyrin repeat region; SH3, Src homology 3 domain; PDZ, PSD-95/Dlg/ZO-1 domain; Proline-rich, proline-rich region; SAM, sterile alpha motif domain. **(B)** A schematic diagram showing the gene-targeting strategy used to generate *Shank3*^Q321R^ mice. Note that the mutated codon corresponding to the Q321R mutation is located in exon 8 of the *Shank3* gene. Primers (forward and reverse) for PCR genotyping are indicated. Neo, neomycin resistance; DTA, diphtheria toxin. **(C)** Confirmation of the *Shank3*^Q321R^ mutation by genomic DNA sequencing of *Shank3*^Q321R/Q321R^ mice. Results from a wild-type (WT) mouse are shown for comparison. **(D)** PCR genotyping of *Shank3*^Q321R/Q321R^ (HM/homozygous) and *Shank3*^+/Q321R^ (HT/heterozygous) mice. **(E)** Known Shank3 protein variants. The target region (aa 1431–1590) of the Shank3 antibody used in the immunoblot analysis **(F)** is indicated. **(F)** The levels of Shank3a, but not Shank3c/d or Shank3e, protein variants, are substantially decreased in whole-brain lysates from WT and *Shank3*^Q321R/Q321R^ and *Shank3*^+/Q321R^ mice (2 months; female), as determined by immunoblot analysis using Shank3-specific antibodies targeting amino acids 1431–1590 in the proline-rich region of the protein that can detect all three protein splice variants. *n* = 3 mice (WT, HT, and HM), ^∗^*P* < 0.05, ^∗∗^*P* < 0.01, ns, not significant, One sample *t*-test. **(G)** Normal gross morphology of the brains of WT and homozygous *Shank3*^Q321R/Q321R^ mice (2 months; female), as shown by immunostaining of coronal sections for the neuronal marker, NeuN. Scale bar, 1 mm.

To determine *in vivo* impacts of the Shank3 Q321R mutation, we generated a new knock-in (KI) mouse line carrying this mutation (*Shank3*^Q321R^ mice) (Figure 2A,B). DNA sequencing confirmed the *Shank3*^Q321R^ mutation (Figure 2C), and wild-type (WT) and *Shank3*^Q321R^ mice (heterozygous *Shank3*^+/Q321R^ and homozygous *Shank3*^Q321R/Q321R^) were detected by polymerase chain reaction (PCR) genotyping (Figure 2D).

The mutated codon corresponding to the Shank3 Q321R mutation is located in exon 8 of the *Shank3* gene, which encodes the C-terminal region of the ARR domain of the Shank3 protein. Therefore, among the multiple variants of the Shank3 protein, Shank3a and Shank3b, containing the ARR domain (Figure 2E), would be most strongly affected compared with ARR-lacking Shank3c/d/e variants (Lim et al., 1999; Maunakea et al., 2010; Wang et al., 2011, 2014; Waga et al., 2014; Zhu et al., 2014). Indeed, the levels of Shank3a, the longest (∼240 kDa) variant, which is readily detected by an antibody targeting the proline-rich region (aa 1431–1590), were significantly decreased (by ∼18%) in homozygous *Shank3*^Q321R/Q321R^ brains (Figure 2F), in line with the predicted destabilization of the Shank3 protein noted above. Detection of Shank3b protein, which lacks a large portion of the proline-rich region, was not possible because the antibody used targets the proline-rich region. In heterozygous *Shank3*^+/Q321R^ brains, Shank3a levels were comparable to those in WT mice, indicative of less severe protein degradation of the mutant Shank3 protein in the heterozygous *Shank3*-mutant brain.

*Shank3*^Q321R/Q321R^ and *Shank3*^+/Q321R^ mice were born in the expected Mendelian ratios. In addition, *Shank3*^Q321R/Q321R^ mice displayed no detectable abnormalities in the size or gross morphology of the brain, as shown by staining for the neuronal marker, NeuN (Figure 2G).

### Suppressed Neuronal Excitability in Homozygous *Shank3*^Q321R/Q321R^ Hippocampal CA1 Pyramidal Neurons

**FIGURE 3:**
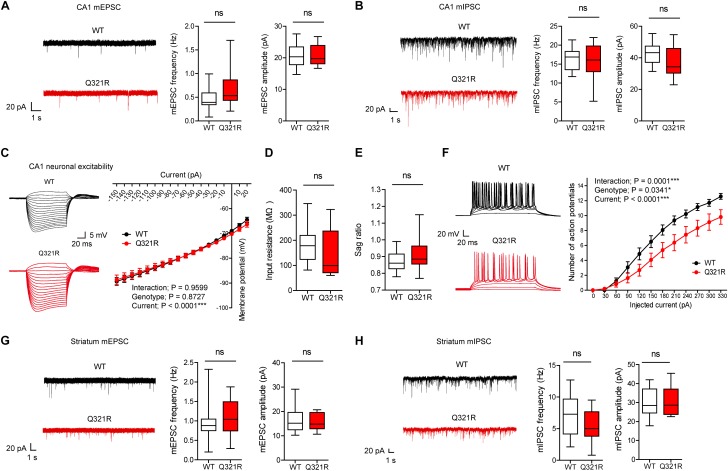
Suppressed neuronal excitability but normal mEPSCs and mIPSCs in hippocampal CA1 pyramidal neurons from male *Shank3*^Q321R/Q321R^ mice. **(A)** Normal mEPSC frequency and amplitude in CA1 pyramidal neurons in the hippocampus of male *Shank3*^Q321R/Q321R^ mice (P21–25). The indicated values represent means ± SEM. *n* = 14 neurons from 3 mice (WT) and 15 neurons from 3 mice (Q321R), ns, not significant, Mann–Whitney *U*-test (for frequency) and Student’s *t*-test (for amplitude). **(B)** Normal mIPSC frequency and amplitude in CA1 pyramidal neurons in the hippocampus of male *Shank3*^Q321R/Q321R^ mice (P21–25). *n* = 12 neurons from 5 mice (WT) and 13 neurons from 3 mice (Q321R), ns, not significant, Student’s *t*-test (for frequency) and Student’s *t*-test (for amplitude). **(C–F)** Suppressed neuronal excitability in hippocampal CA1 pyramidal neurons from male *Shank3*^Q321R/Q321R^ mice (P22–26), as shown by the number of action potential fired plotted against injected currents **(F)**. Note that the current-voltage relationship **(C)**, input resistance **(D)**, and Sag ratio **(E)** were not changed. *n* = 14 neurons from 5 mice (WT) and 16 neurons from 4 mice (Q321R), ^∗^*P* < 0.05, ^∗∗∗^*P* < 0.001, ns, not significant, repeated measures two-way ANOVA (for current-membrane potential and current-firing curves) and Mann–Whitney *U*-test (for input resistance and sag ratio). **(G)** Normal mEPSC frequency and amplitude in dorsolateral striatal neurons from male *Shank3*^Q321R/Q321R^ mice (P28–43). *n* = 16 neurons from 4 mice (WT) and 16 neurons from 5 mice (Q321R), ns, not significant, Mann–Whitney *U*-test (for frequency) and Student’s *t*-test (for amplitude). **(H)** Normal mIPSC frequency and amplitude in dorsolateral striatal neurons from male *Shank3*^Q321R/Q321R^ mice (P28–43). *n* = 16 neurons from 6 mice (WT) and 17 neurons from 7 mice (Q321R), ns, not significant, Student’s *t*-test (for frequency) and Mann–Whitney *U*-test (for amplitude).

To determine the effect of the Shank3 Q321R mutation on synaptic transmission and neuronal excitability, we first measured spontaneous synaptic transmission in the hippocampus, a brain region implicated in ASD (Amaral et al., 2008). The frequency and amplitude of miniature excitatory postsynaptic currents (mEPSCs) in CA1 pyramidal neurons from the hippocampus of homozygous *Shank3*^Q321R/Q321R^ mice (P21–25; male) were comparable to those from WT neurons (Figure 3A). Similarly, the frequency and amplitude of miniature inhibitory postsynaptic currents (mIPSCs) were normal in hippocampal CA1 pyramidal neurons from male *Shank3*^Q321R/Q321R^ mice (P21–25; male) (Figure 3B).

When female mice were tested for mEPSCs and mIPSCs, the frequency, but not amplitude, of mIPSCs was increased in CA1 pyramidal neurons (P22–26), whereas mEPSC frequency and amplitude in these neurons (P21–27) were normal (Supplementary Figures S1A,B). Given that Shank proteins are mainly present at excitatory, but not inhibitory, synapses (Boeckers et al., 1999; Naisbitt et al., 1999; Tu et al., 1999; Yao et al., 1999; Bockmann et al., 2002; Heise et al., 2016), these results likely represent a secondary change rather than the direct consequence of *Shank3* deletion in pyramidal neurons.

We next measured the neuronal excitability of CA1 pyramidal neurons from *Shank3*^Q321R/Q321R^ mice (P22–26; male). Neuronal excitability was decreased, as evidenced by the relationship between the amount of injected current and the number of action potentials fired, but the current-voltage relationship, input resistance, and Sag ratio were normal (Figure 3C–F). These results collectively suggest that the Shank3 Q321R mutation suppresses neuronal excitability in male CA1 pyramidal neurons, which would suppress the output function of these neurons.

In addition to the hippocampus, we measured mEPSCs and mIPSCs in the striatum, a brain region strongly implicated in the pathophysiology of Shank3-related autistic-like phenotypes in mice (Peca et al., 2011; Peixoto et al., 2016; Lee et al., 2017). We found that the frequency and amplitude of mEPSCs in dorsolateral striatal neurons in *Shank3*^Q321R/Q321R^ mice (P28–43; male) were normal (Figure 3G). In addition, mIPSC frequency and amplitude were normal in these neurons (P28–43; male) (Figure 3H). These results suggest that the *Shank3* Q321R homozygous mutation does not affect spontaneous excitatory or inhibitory synaptic transmission in the dorsolateral striatum in male mice.

### Normal Locomotion and Moderate Anxiolytic-Like Behavior in Heterozygous *Shank3*^+/Q321R^ Mice

To determine the behavioral impacts of the Shank3 Q321R mutation, we next subjected *Shank3*^+/Q321R^ and *Shank3*^Q321R/Q321R^ mice to a battery of behavioral tests. Previous studies on *Shank3*-mutant mice have shown that *Shank3* heterozygosity can lead to ASD-related behavioral abnormalities (Bozdagi et al., 2010; Yang et al., 2012; Duffney et al., 2015; Jaramillo et al., 2016, 2017). We thus first analyzed behavioral abnormalities in heterozygous *Shank3*^+/Q321R^ mice.

**FIGURE 4:**
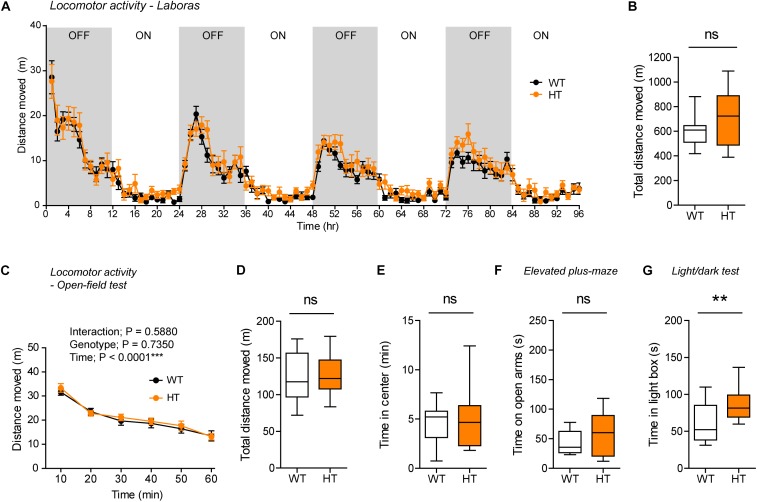
Normal locomotor activity and moderate anxiolytic-like behavior in heterozygous *Shank3*^+/Q321R^ mice. **(A,B)** Normal locomotor activity in *Shank3*^+/Q321R^ mice (2–3 months; male) in Laboras cages, where locomotor activity was measured together with other movements for four consecutive days in the absence of habituation. OFF/ON, light-off/on. *n* = 13 mice (WT) and 13 mice (HT), ns, not significant, two-way ANOVA (genotype main effect *p*-value = 0.1813 in **A**), and Student’s *t*-test **(B)**. **(C,D)** Normal locomotor activity in *Shank3*^+/Q321R^ mice (2–3 months; male) in the open-field test, as shown by the distance moved. *n* = 13 mice (WT) and 13 mice (HT), ns, not significant, repeated measures two-way ANOVA **(C)** and Student’s *t*-test **(D)**. **(E)** Normal anxiety-like behavior in *Shank3*^+/Q321R^ mice (2–3 months; male) in the open-field test, as shown by the time spent in the center region of the open-field arena. *n* = 13 mice (WT) and 13 mice (HT), ns, not significant, Mann–Whitney *U*-test. **(F)** Normal anxiety-like behavior in *Shank3*^+/Q321R^ mice (2–3 months; male) in the elevated plus-maze test, as shown by the time spent in the open arms of the maze. *n* = 13 mice (WT) and 13 mice (HT), ns, not significant, Welch’s *t*-test. **(G)** Anxiolytic-like behavior in *Shank3*^+/Q321R^ mice (2–3 months; male) in the light-dark test, as shown by the time spent in the light chamber of the light-dark apparatus. *n* = 13 mice (WT) and 12 mice (HT), ^∗∗^*P* < 0.01, Student’s *t*-test.

Continuous monitoring of mouse movements for four consecutive days in Laboras cages, representing a familiar environment, revealed that *Shank3*^+/Q321R^ mice have normal locomotor activity compared with WT mice (Figure 4A,B). Similarly, *Shank3*^+/Q321R^ mice displayed normal locomotor activity in the open-field test, a novel environment (Figure 4C,D). These results suggest that locomotor activities in both familiar and novel environments are not affected by the heterozygous Shank3 Q321R mutation.

*Shank3*^+/Q321R^ and WT mice spent comparable amounts of time in the center region of the open-field arena (Figure 4E). In addition, *Shank3*^+/Q321R^ mice spent normal amounts of time in the open arms of the EPM (Figure 4F). In contrast, these mice spent more time in the light chamber of the light-dark apparatus (Figure 4G). Together, these results suggest that the heterozygous Shank3 Q321R mutation leads to moderate anxiolytic-like behavior but does not affect locomotor activity.

### Normal Social Interaction, Moderately Enhanced Social Communication and Self-Grooming, and Suppressed Digging in Heterozygous *Shank3*^+/Q321R^ Mice

**FIGURE 5:**
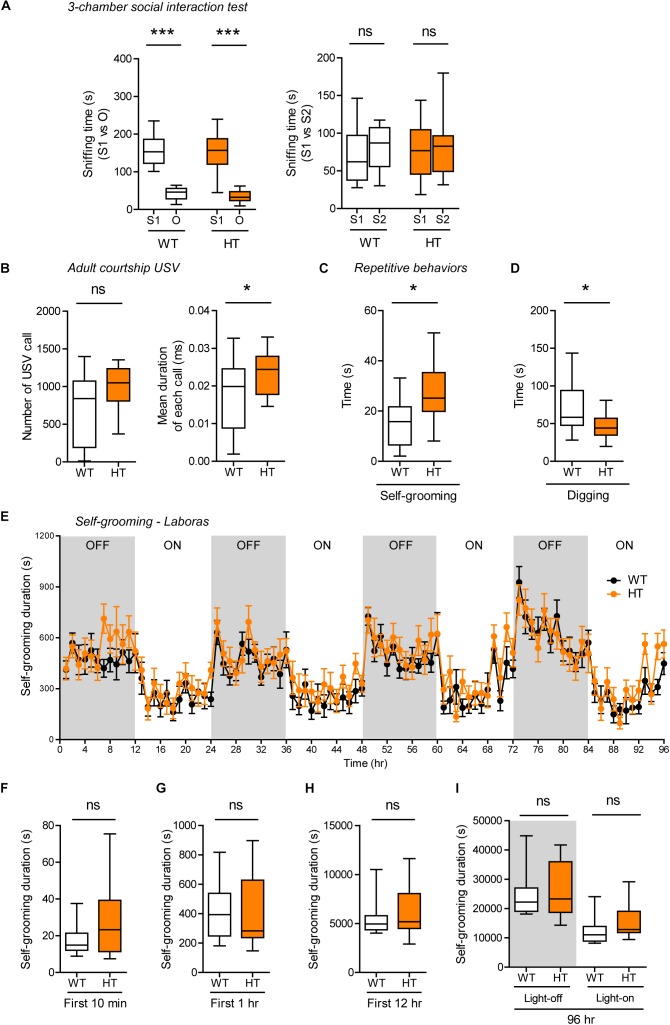
Normal social interaction, moderately enhanced social communication and self-grooming, and suppressed digging in heterozygous *Shank3*^+/Q321R^ mice. **(A)** Normal social approach in *Shank3*^+/Q321R^ mice (2–3 months; male) in the three-chamber test, as shown by time spent sniffing. S1, a stranger; O, object; S2, new stranger. Social novelty recognition, measured by the preference for new stranger (S2) over old stranger (S1), could not be determined due the lack of normal social novelty recognition in WT mice. *n* = 10 mice (WT), 13 mice (HT), ^∗∗∗^*P* < 0.001, ns, not significant, Welch’s *t*-test, Mann–Whitney *U*-test, and Student’s *t*-test. **(B)** Moderately increased courtship USVs emitted by *Shank3*^+/Q321R^ mice (2–3 months; male) upon encounter with a novel female stranger, as shown by the normal number of USVs but increased mean duration of each USV calls. *n* = 12 mice (WT), 12 mice (HT), ^∗^*P* < 0.05, ns, not significant, Student’s *t*-test. **(C)** Enhanced self-grooming in *Shank3*^+/Q321R^ mice (2–3 months; male) in home cages with bedding (10 min), as shown by total self-grooming time. *n* = 12 mice (WT), 13 mice (HT), ^∗^*P* < 0.05, Student’s *t*-test. **(D)** Suppressed digging in *Shank3*^+/Q321R^ mice (2–3 months; male) in home cages with bedding (10 min), as shown by total digging time. *n* = 12 mice (WT), 13 mice (HT), ^∗^*P* < 0.05, Welch’s *t*-test. **(E–I)** Normal self-grooming in *Shank3*^+/Q321R^ mice (2–3 months; male) in Laboras cages, as shown by total self-grooming duration. *n* = 13 mice (WT), 13 mice (HT), ns, not significant, two-way ANOVA (genotype main effect *p*-value = 0.4087 in **E**), Mann-Whitney test **(F,H,I)**, Student’s *t*-test **(G)**.

*Shank3*^+/Q321R^ and WT mice showed comparable levels of social approach in the three-chamber test, as shown by time spent sniffing social and object targets (Figure 5A). Social novelty recognition could not be assessed because WT mice failed to recognize a novel stranger mouse. In tests for USVs in adult male mice encountering a novel female mouse (courtship USVs), *Shank3*^+/Q321R^ mice emitted normal numbers of USVs, but the mean duration of each call was increased (Figure 5B), indicative of a moderate and abnormal increase in social communication.

In tests for repetitive behaviors, *Shank3*^+/Q321R^ mice displayed enhanced self-grooming and suppressed digging in novel home cages with bedding (10 min; ∼60 lux) (Figure 5C,D), suggesting that the suppressed digging might result from the enhanced self-grooming. However, long-term (96-h) monitoring of behavior in Laboras cages showed that *Shank3*^+/Q321R^ mice exhibit normal self-grooming during the first 10 min, the first 1 or 12 h, the entire session (96 h), and during light-off and light-on periods (48 h each) (Figure 5E–I). These results collectively suggest that the heterozygous Shank3 Q321R mutation does not affect social approach, moderately enhances social communication and self-grooming, and suppresses digging in mice.

### Homozygous *Shank3*^Q321R/Q321R^ Mice Show Behaviors That Are Largely Similar to Those Observed in Heterozygous *Shank3*^+/Q321R^ Mice

**FIGURE 6:**
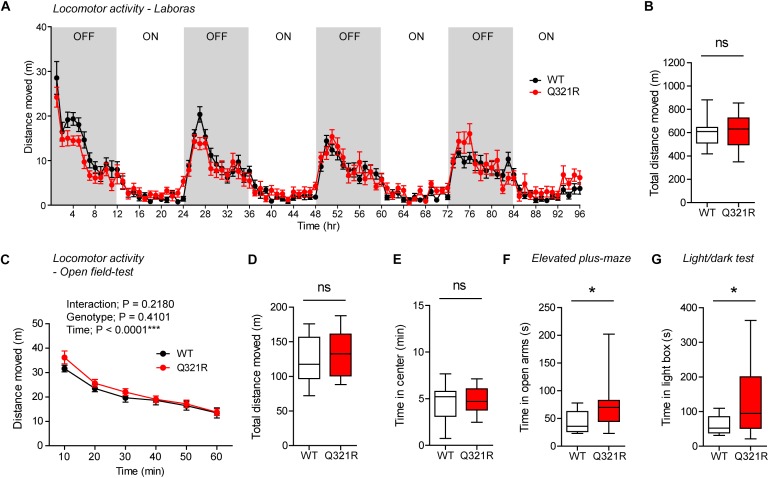
Normal locomotor activity and moderate anxiolytic-like behavior in homozygous *Shank3*^Q321R/Q321R^ mice. **(A,B)** Normal locomotor activity in *Shank3*^Q321R/Q321R^ mice (2–4 months; male), in Laboras cages, where locomotor activity was measured together with other movements for four consecutive days in the absence of habituation. Note that the WT data in this panel and other panels in this figure are identical to those shown in Figure 4 because WT, heterozygous, and homozygous mice were tested together in the same behavioral tests. OFF/ON, light-off/on. *n* = 13 mice (WT) and 14 mice (Q321R), ns, not significant, two-way ANOVA (**A**, genotype main effect *p*-value = 0.9356) and Student’s *t*-test **(B)**. **(C,D)** Normal locomotor activity in *Shank3*^Q321R/Q321R^ mice (2 months; male) in the open-field test, as shown by the distance moved. Note that *Shank3*^Q321R^ mice spent a normal amount of time in the center region of the open-field arena, suggestive of normal anxiety-like behavior. *n* = 13 mice (WT) and 14 mice (Q321R), ^∗∗∗^*P* < 0.001, ns, not significant, repeated measures two-way ANOVA **(C)** and Student’s *t*-test **(D)**. **(E)** Normal anxiety-like behavior in *Shank3*^Q321R/Q321R^ mice (2 months; male) in the open-field test, as shown by the time spent in the center region of the open-field arena. *n* = 13 mice (WT) and 14 mice (Q321R), ns, not significant, Student’s *t*-test. **(F)** Anxiolytic-like behavior in *Shank3*^Q321R/Q321R^ mice (2 months; male) in the elevated plus-maze test, as shown by the increased time spent in the open arms of the maze. *n* = 13 mice (WT) and 14 mice (Q321R), ^∗^*P* < 0.05, Mann–Whitney *U*-test. **(G)** Anxiolytic-like behavior in *Shank3*^Q321R/Q321R^ mice (2 months; male) in the light-dark test, as shown by the increased time spent in the light chamber of the light-dark apparatus. *n* = 13 mice (WT) and 14 mice (Q321R), ^∗^*P* < 0.05, Welch’s *t*-test.

To test whether there are any dose-dependent effects of the Shank3 Q321R mutation on mouse behaviors, we subjected homozygous *Shank3*^Q321R/Q321R^ mice to a battery of behavioral tests that were used for heterozygous *Shank3*^+/Q321R^ mice. *Shank3*^Q321R/Q321R^ mice showed normal levels of locomotor activity in Laboras cages and in the open-field test (Figure 6A–D). In addition, these mice showed moderately increased anxiolytic-like behaviors, as shown by normal levels of center time in the open-field test but increased open-arm time in the EPM and increased light-box time in the light-dark test (Figure 6E–G), similar to heterozygous *Shank3*^+/Q321R^ mice.

**FIGURE 7:**
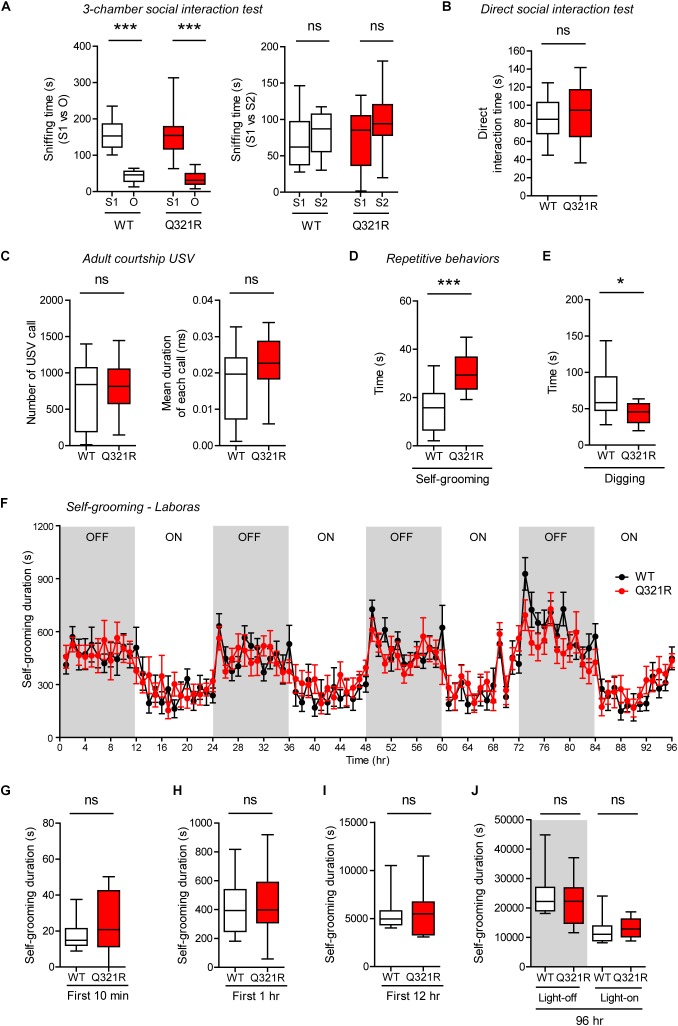
Normal social interaction and communication, moderately enhanced stress-induced self-grooming, and suppressed digging in homozygous *Shank3*^Q321R/Q321R^ mice. **(A)** Normal social approach in *Shank3*^Q321R/Q321R^ mice (3 months; male) in the three-chamber test, as shown by time spent sniffing social (S1) or object (O) target. Social novelty recognition, measured by the preference for new stranger (S2) over old stranger (S1), could not be determined due the lack of normal social novelty recognition in WT mice. Note that the WT data in this panel and other panels in this figure are identical to those shown in Figure 5 because WT, heterozygous, and homozygous mice were tested together in the same behavioral tests. *n* = 10 mice (WT), 14 mice (Q321R), ^∗∗∗^*P* < 0.001, ns, not significant, Welch’s *t*-test (for S1 vs. O) and Student’s *t*-test (for S1 vs. S2). **(B)** Normal social interaction in *Shank3*^Q321R/Q321R^ mice (3 months; male) in bidirectional direct social-interaction tests, as shown by total time spent in social interaction. *n* = 10 pairs (WT), 13 pairs (Q321R), ns, not significant, Student’s *t*-test. **(C)** Normal USVs emitted by *Shank3*^Q321R/Q321R^ mice (4 months; male) upon encounter with a novel female stranger (courtship USVs), as shown by the total number of USVs and the mean duration of each USV calls. *n* = 12 mice (WT), 14 mice (Q321R), ns, not significant, Student’s *t*-test. **(D)** Enhanced self-grooming in *Shank3*^Q321R/Q321R^ mice (2 months; male) in home cages with bedding (10 min), as shown by total self-grooming time. *n* = 12 mice (WT), 13 mice (Q321R), ^∗∗∗^*P* < 0.001, Student’s *t*-test. **(E)** Suppressed digging in *Shank3*^Q321R/Q321R^ mice (2 months; male) in home cages with bedding (10 min), as shown by total digging time. *n* = 12 mice (WT), 13 mice (Q321R), ^∗^*P* < 0.05, Welch’s *t*-test. **(F–J)** Normal self-grooming in *Shank3*^Q321R/Q321R^ mice (2 months; male) in Laboras cages, where self-grooming was measured together with other movements for four consecutive days in the absence of habituation. OFF/ON, light-off/on. Note that there are no genotype differences during the first 10 min, 1 or 12 h, the entire session (96 h), or during light-off and light-on periods (48 h each). *n* = 13 mice (WT) and 14 mice (Q321R), ns, not significant, two-way ANOVA (genotype main effect *p*-value = 0.9754 in **F**), Student’s *t*-test **(H)** and Mann–Whitney *U*-test **(G,I,J)**.

Social behavior was normal in homozygous *Shank3*^Q321R/Q321R^ mice, as shown by the three-chamber test (Figure 7A), similar to heterozygous *Shank3*^+/Q321R^ mice. Again, social novelty recognition could not be assessed because WT mice failed to recognize a novel stranger mouse. *Shank3*^Q321R/Q321R^ and WT mice showed comparable levels of social interaction in the direct social interaction test, as shown by the total time spent interacting with an age- and sex-matched male stranger mouse (Figure 7B).

Homozygous *Shank3*^Q321R/Q321R^ mice displayed normal courtship USVs, as shown by the number of USV calls as well as the duration of each USV calls (Figure 7C), partly similar to heterozygous *Shank3*^+/Q321R^ mice that displayed a normal number of courtship USV calls but increased duration of each USV calls. Lastly, homozygous *Shank3*^Q321R/Q321R^ mice displayed increased self-grooming and decreased digging in novel home cages with bedding (10 min) (Figure 7D,E), but normal Laboras-cage self-grooming (Figure 7F–J), largely similar to heterozygous *Shank3*^+/Q321R^ mice.

Because the differences between novel home-cage and Laboras-cage environments, where both *Shank3*^+/Q321R^ and *Shank3*^Q321R/Q321R^ mice showed positive and negative repetitive self-grooming, respectively, include the novelty of the space (less novel in new home cages and more novel in Laboras cages) and the light intensity (bright light in home cages and complete darkness in Laboras cages). To differentiate these factors, we first tested a new condition, novel home-cage environment without light, and, intriguingly, could not observe enhanced self-grooming in *Shank3*^Q321R/Q321R^ mice (Supplementary Figure 2A), suggesting that the presence of light is important. However, the presence of light in Laboras cages did not enhance self-grooming in *Shank3*^Q321R/Q321R^ mice during the first 10 min (Supplementary Figure 2B), similar to the results from Laboras cages without light. Furthermore, the presence of light in a novel open-field arena did not enhance self-grooming in *Shank3*^Q321R/Q321R^ mice (Supplementary Figure 2C). These results indicate that self-grooming in *Shank3*^Q321R/Q321R^ mice is enhanced selectively in novel home cages in the presence of light. In addition, these results suggest that novelty of space/environment *per se* is not important, but, rather, an increase in stress associated with the light in novel home cages (but not in other environments) strongly enhances self-grooming in *Shank3*^Q321R/Q321R^ mice.

Together, these results suggest that homozygous *Shank3*^Q321R/Q321R^ mice show behavioral abnormalities that are largely similar to those observed in heterozygous *Shank3*^+/Q321R^ mice such as moderately enhanced anxiolytic-like behavior and self-grooming, although there were minor differences in a sub-parameter of USVs (duration of each USV calls). In addition, the largely similar behaviors of *Shank3*^+/Q321R^ and *Shank3*^Q321R/Q321R^ mice suggest that there is no strong dose-dependent effect of the Shank3 Q321R mutation on mouse behaviors.

### Normal Novel Object-Recognition and Contextual Fear Memory in Homozygous *Shank3*^Q321R/Q321R^ Mice

**FIGURE 8:**
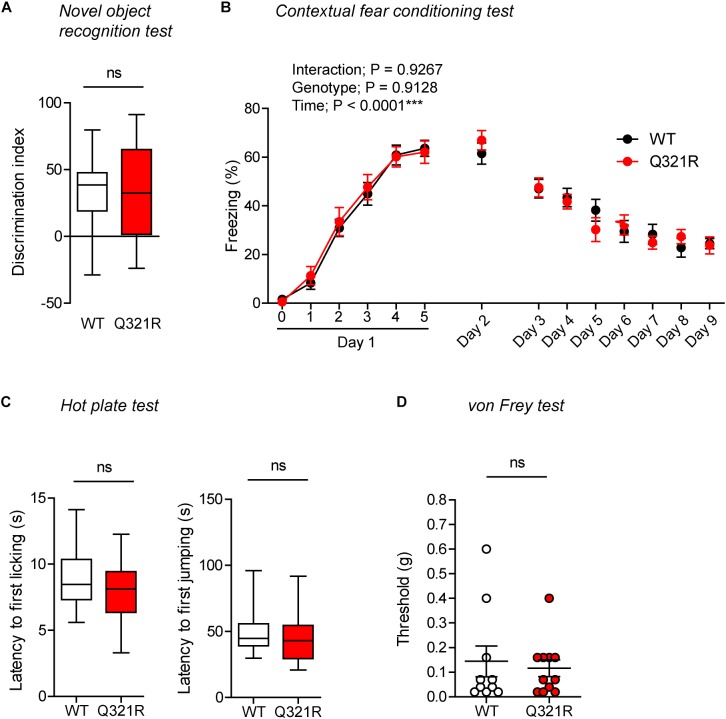
Normal object-recognition and fear memory in homozygous male *Shank3*^Q321R/Q321R^ mice. **(A)** Normal levels of object recognition memory in *Shank3*^Q321R/Q321R^ mice (2–4 months; male) in the novel object recognition test, as shown by the discrimination index over a familiar object and a novel object (see Materials and Methods for details) presented 24 h after exploring two identical objects on the first day. *n* = 11 mice (WT), 13 mice (Q321R), ns, not significant, Student’s *t*-test. **(B)** Normal acquisition, retrieval, and extinction of contextual fear memory *Shank3*^Q321R/Q321R^ mice (3–6 months; male), as shown by freezing levels. Mice were given 5 foot shocks (2-min intervals) during the 12-min fear memory acquisition phase (day 1), and were consecutively exposed to the same context 24 h after the training (day 2) and also during days 3–9 for fear extinction. *n* = 13 mice (WT), 17 mice (Q321R), repeated measures two-way ANOVA. **(C)** Normal somatosensory function in *Shank3*^Q321R/Q321R^ mice (6 months; male) in the hot plate test, as shown by latency to first licking/jumping. *n* = 17 mice (WT) and 20 mice (Q321R), ns, not significant, Student’s *t*-test (for latency to first licking), and Mann–Whitney *U*-test (for latency to first jumping). **(D)** Normal somatosensory function in *Shank3*^Q321R/Q321R^ mice (2 months; male) in the von Frey test, as shown by threshold for response to stimulation. *n* = 10 mice (WT) and 11 mice (Q321R), ns, not significant, Mann–Whitney *U*-test.

In the novel object–recognition test, in which a mouse is exposed to two identical objects on day 1 and then to one original object and a new object on day 2, *Shank3*^Q321R/Q321R^ and WT mice showed similar discrimination index scores for the novel object (Figure 8A), indicative of normal object-recognition memory in the mutant mice.

In the contextual fear-conditioning test, *Shank3*^Q321R/Q321R^ mice showed normal acquisition of fear memory on the training day (day 1) (Figure 8B). Twenty-four hours later (day 2), *Shank3*^Q321R/Q321R^ and WT mice showed comparable levels of freezing in the same context, suggestive of normal retrieval of contextual fear memory. When these mice were subsequently exposed to the same context every day for seven consecutive days (day 3–9) for fear extinction, they displayed comparable decreases in freezing levels, suggestive of normal fear extinction in *Shank3*^Q321R/Q321R^ mice.

In addition, *Shank3*^Q321R/Q321R^ mice showed normal levels of somatosensory functions, as determined by the hot plate and von Frey tests (Figure 8C,D). These results collectively suggest that the homozygous Shank3 Q321R mutation does not affect object-recognition memory, acquisition, or extinction of contextual fear memory, or somatosensory functions.

### Female Homozygous *Shank3*^Q321R/Q321R^ Mice Show Normal Anxiety-Like Behavior and Reduced Self-Grooming

To test whether there is a male-female difference in the impacts of the Shank3 Q321R mutation on behaviors, we subjected female *Shank3*^Q321R/Q321R^ mice to the behavioral tests in which male *Shank3*^Q321R/Q321R^ mice showed abnormal behaviors. To our surprise, female *Shank3*^Q321R/Q321R^ mice showed normal levels of anxiety-like behaviors in both EPM and light-dark tests (Supplementary Figures 1C,D), dissimilar to male *Shank3*^Q321R/Q321R^ mice that showed anxiolytic-like behavior in both tests (Figure 6F,G).

Furthermore, female *Shank3*^Q321R/Q321R^ mice showed normal levels of self-grooming and digging in novel home cages with bedding (10 min) (Supplementary Figures 1E,F), again dissimilar to male *Shank3*^Q321R/Q321R^ mice that showed enhanced self-grooming and reduced digging in novel home cages (Figure 7D,E). These results collectively suggest that female *Shank3*^Q321R/Q321R^ mice do not show anxiolytic-like behavior or enhanced self-grooming, dissimilar to male *Shank3*^Q321R/Q321R^ mice.

### Abnormal EEG Patterns and Decreased Susceptibility to Induced Seizures in Homozygous Male *Shank3*^Q321R/Q321R^ Mice

**FIGURE 9:**
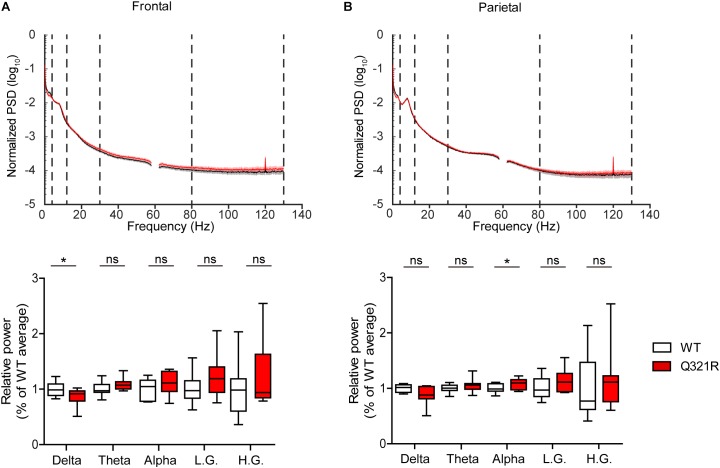
Abnormal EEG patterns in *Shank3*^Q321R/Q321R^ mice. **(A,B)** Abnormal EEG patterns in frontal and parietal lobes of the *Shank3*^Q321R/Q321R^ brain (3 months; male). EEG power was calculated by combining data from both left and right hemispheres in the frontal or parietal lobe. Note that the EEG power in the delta frequency range is decreased, whereas that in the alpha frequency range is increased. The vertical dotted lines in the power spectral density (PSD) diagrams indicate the boundaries between different frequency ranges (delta, 0–4 Hz; theta, 4–12 Hz; alpha, 12–30 Hz; low gamma, 30–80 Hz; high gamma, 80–130 Hz). *n* = 10 mice (WT) and 10 mice (Q321R) for frontal lobe and 9 mice (WT) and 10 mice (Q321R) for parietal lobe, ^∗^*P* < 0.05, ns, not significant, Student’s *t*-test, Mann–Whitney *U*-test, Welch’s *t*-test.

Abnormal electroencephalogram (EEG) patterns have been observed in ASD (Wang et al., 2013), ASD and PMS associated with *SHANK3* mutations (Moessner et al., 2007; Soorya et al., 2013; Figura et al., 2014; Holder and Quach, 2016), as well as in *Shank3*-mutant mouse models (Han et al., 2013; Dhamne et al., 2017). In particular, the individual with ASD carrying the Q321R mutation was reported to display abnormal EEG, bilateral epileptiform discharges without seizures, and severe sleep disorders (Moessner et al., 2007). We thus attempted bilateral EEG recordings in the frontal and parietal lobes of the male *Shank3*^Q321R/Q321R^ brain using EEG drivers implanted on the skull. The power of EEG in the delta range, but not other frequency ranges (0–4 Hz), was decreased in the frontal lobes, whereas EEG power in the alpha frequency range (12–30 Hz) was increased in the parietal lobes (Figure 9A,B).

Abnormalities in the left-right hemispheric asymmetry of EEGs have also been observed in ASD (Wang et al., 2013). We thus analyzed the changes in EEGs in left and right hemispheres of the *Shank3*^Q321R/Q321R^ brain separately. In the frontal lobe, left and right hemispheres showed similar decreases in normalized EEG power in the delta range, but not other ranges (Supplementary Figure 3A). Intriguingly, in the parietal lobe, the right but not left hemisphere showed decreased EEG power in the delta range and increased EEG power in theta and alpha ranges (Supplementary Figure 3B). These results suggest that the decreased delta-band EEG power in the frontal lobe likely involves both hemispheres, whereas the increase alpha-band EEG power in the parietal lobe likely involves the right parietal lobe.

These abnormal EEG patterns in the *Shank3*^Q321R/Q321R^ brain and reduced neuronal excitability in *Shank3*^Q321R/Q321R^ hippocampal CA1 neurons (Figure 3) suggest the possibility that the *Shank3*^Q321R/Q321R^ mutation disrupts the balance between neuronal excitation and inhibition in the brain, a mechanism suggested to underlie ASD (Rubenstein and Merzenich, 2003). To test this, we assessed the susceptibility of *Shank3*^Q321R/Q321R^ mice to seizures induced by the GABA_A_ receptor antagonist pentylenetetrazole (PTZ).

**FIGURE 10:**
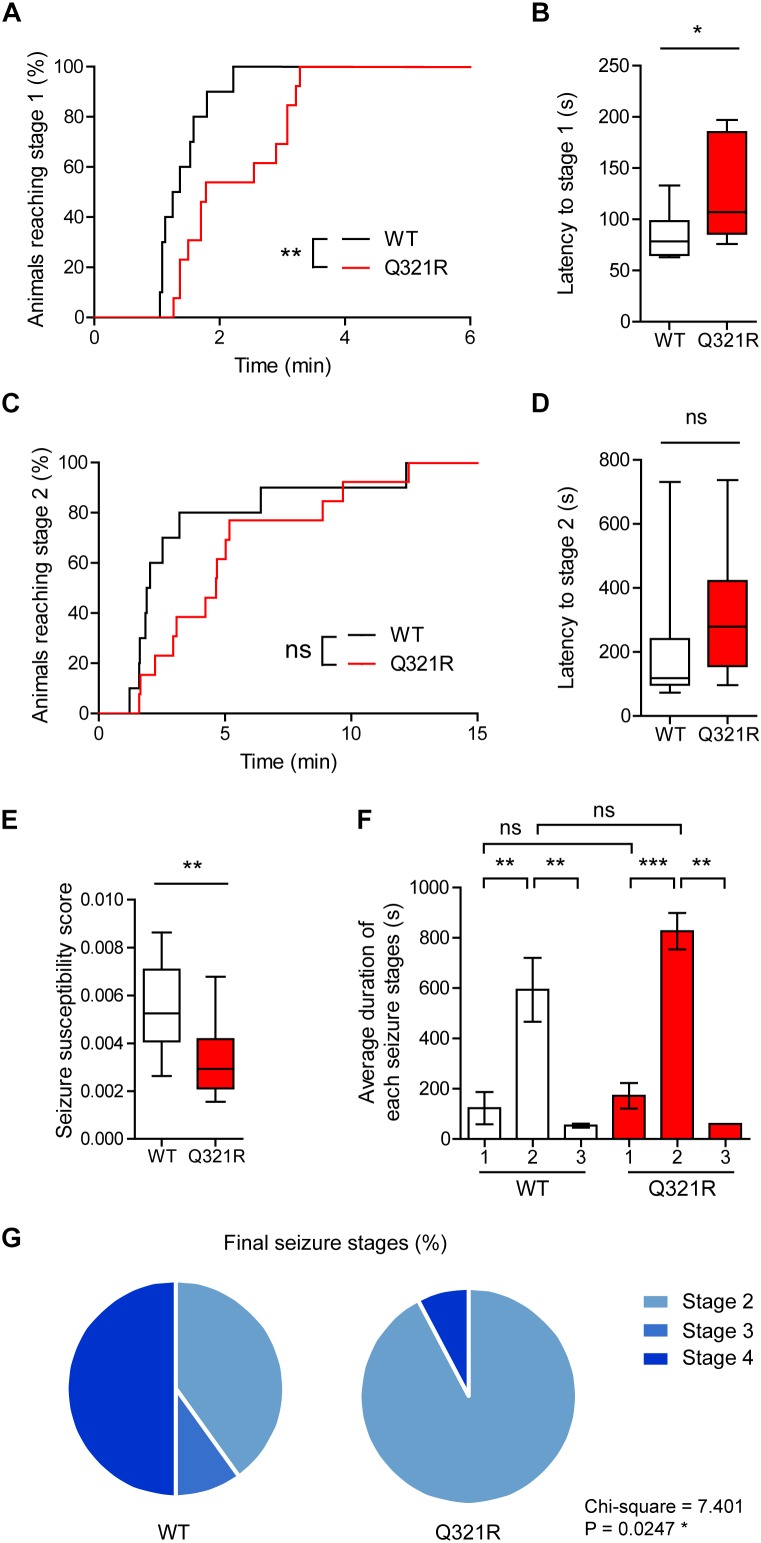
Decreased susceptibility to PTZ-induced seizures in *Shank3*^Q321R/Q321R^ mice. **(A)** Percentage of animals that can reach stage 1 (behavioral arrest) seizures. Seizures were induced in WT and *Shank3*^Q321R/Q321R^ mice (3–4 months; male) by intraperitoneal injection of PTZ (50 mg/kg). *n* = 10 mice (WT), 13 mice (Q321R), ^∗∗^*P* < 0.01, log-rank (Mantel-Cox) test (*p* = 0.0047). **(B)** Latency to reach stage 1 (behavioral arrest) seizures. ^∗^*P* < 0.05, Mann–Whitney *U*-test. **(C)** Percentage of animals that can reach stage 2 (myoclonic) seizures. ns, not significant, log-rank (Mantel-Cox) test (*p* = 0.1881). **(D)** Latency to reach stage 2 (myoclonic) seizures. ns, not significant, Mann–Whitney *U*-test. **(E)** Seizure susceptibility based on the latencies to reach stage 1, 2, or 3 seizure (see Materials and Methods for details on the definition of seizure susceptibility). ^∗∗^*P* < 0.01, Student’s *t*-test. **(F)** Duration of seizure stages 1, 2, and 3. ^∗∗^*P* < 0.01, ^∗∗∗^*P* < 0.001, ns, not significant, one-way ANOVA with Kruskal–Wallis/Holm–Sidak test (within-genotype stage 1/2/3 comparison) and Mann–Whitney *U*-test (between-genotype stage 1/2/3 comparison). **(G)** Final seizure stages that animals reached, expressed as a percentage of animals. ^∗^*P* < 0.05, Chi-square test.

We found that male *Shank3*^Q321R/Q321R^ mice are less susceptible to PTZ-induced seizures compared with WT mice, as shown by the latency to seizure stage 1 (although not stage 2), latency-based seizure susceptibility index, and final seizure stages reached (Figure 10). Spontaneous behavioral seizures were not observed in the absence of PTZ injection. These results collectively suggest that the homozygous Shank3 Q321R mutation induces abnormal EEG patterns and decreased PTZ-induced seizure susceptibility in male mice.

## Discussion

Our results indicate that the Shank3 Q321R mutation, located in exon 8 of the *Shank3* gene encoding the C-terminal part of the ARR domain of the protein, leads to substantial destabilization of the ARR-containing Shank3a protein variant and synaptic, behavioral, and EEG/seizure abnormalities in mice.

Our *in vivo* data from *Shank3*^Q321R/Q321R^ mice indicate that the Q321R mutation induces a substantial decrease (∼18% of WT levels) in the total level of the ARR-containing Shank3a protein variant in the *Shank3*^Q321R/Q321R^ brain (Figure 2F). In addition, our molecular modeling analyses suggest that the Q321R mutation is less likely to affect the intramolecular SPN-ARR interaction but more likely to affect the protein stability or intermolecular interactions of Shank3. The ARR domain of Shank3 is known to interact with Sharpin and α-fodrin (Bockers et al., 2001; Lim et al., 2001), and the Q321R mutation has been shown to enhance Shank3 binding to Sharpin but not α-fodrin (Mameza et al., 2013). Given that Shank proteins can be ubiquitinated and deubiquitinated in an activity-dependent manner to regulate excitatory synapse structure and function (Ehlers, 2003; Kerrisk Campbell and Sheng, 2018) and that Sharpin is a component of the E3 ligase complex termed LUBAC (linear ubiquitin chain assembly complex) (Ikeda et al., 2011; Tokunaga et al., 2011; Iwai et al., 2014; Hrdinka and Gyrd-Hansen, 2017), it is tempting to speculate that the strong decrease in the levels of the mutant Shank3 protein (Q321R) might involve enhanced protein ubiquitination. Dissimilar to the results from *Shank3*^Q321R/Q321R^ mice, however, *Shank3*^+/Q321R^ mice show normal levels of Shank3 proteins, as compared with WT mice (Figure 2F). Therefore, distinct pathophysiological mechanisms of the Shank3 Q321R mutation at the protein level (i.e., decreased protein levels vs. abnormal protein function) might lead to similar behavioral abnormalities in *Shank3*^Q321R/Q321R^ and *Shank3*^+/Q321R^ mice, likely by acting on similar sets of neurons in the brain.

Electrophysiologically, there were no changes in the frequency or amplitude of mEPSCs in male *Shank3*^Q321R/Q321R^ CA1 pyramidal or dorsolateral striatal neurons (Figure 3; summarized in Table 1). These results suggest that homozygous deletion of ARR-containing Shank3 protein variants does not affect mEPSCs in hippocampal or striatal neurons. Potential reasons for these results could be that ARR-containing Shank3 protein variants only minimally affect excitatory synapse development and function in hippocampal and striatal neurons or that some compensatory changes have occurred. Similar to our results, mEPSC frequency and amplitude are unchanged in *Shank3* mouse lines carrying similar ARR deletions, including *Shank3*^Δ9^ mice (lacking exon 9) (Lee et al., 2015) and *Shank3*^Δ4–9^ mice (lacking exons 4–9) (Wang et al., 2011; Jaramillo et al., 2016). Notably, however, heterozygous deletion of *Shank3* exons 4–9 in mice induces an increase in mEPSC frequency and a decrease in mEPSC amplitude (Bozdagi et al., 2010), suggesting different effects of homozygous and heterozygous deletions.

**Table 1:** Summary of electrophysiological, behavioral, and brain function phenotypes in *Shank3*^Q321R/Q321R^ mice.

	Subcategory	Measurement	Results
Electrophysiology	Hippocampal CA1 pyramidal neurons	mEPSC	Frequency -, amplitude -
		mIPSC	Frequency -, amplitude -
		Neuronal excitability	↓
	Dorsolateral striatal neurons	mEPSC	Frequency -, amplitude -
		mIPSC	Frequency -, amplitude -
Behavior			
	Social interaction	Three-chamber (social approach)	–
		Direct interaction	–
	Social communication	Adult courtship USV	–
	Repetitive behavior	Self-grooming (Laboras cage)	–
		Self-grooming (novel home cage)	↑
		Digging (novel home cage)	↓
	Locomotor activity	Laboras	–
		Open-field	–
	Anxiety-like behavior	Open field center time	–
		Elevated plus-maze (open-arm time)	↓
		Light/dark box (light-chamber time)	↓
	Learning and memory	Novel object recognition	–
		Contextual fear conditioning, retrieval, and extinction	Acquisition (day 1) - 24-h retrieval (day 2) - Extinction (days 3–9) -
Brain function	EEG	Baseline EEG	Frontal-delta ↓ Parietal-alpha ↑ Left-right asymmetry
	Seizure	Susceptibility to PTZ-induced seizure	↓

*Up and down arrows, increases and decreases, respectively; -, no significant change. Male mice were used to obtain the results listed in the table. Additional measurements for select electrophysiological and behavioral parameters were also made in females and described in Results but not summarized in this table.*

Whereas mEPSCs and mIPSCs were unchanged in male *Shank3*^Q321R/Q321R^ CA1 pyramidal neurons, and mEPSCs were unchanged in female *Shank3*^Q321R/Q321R^ CA1 pyramidal neurons, the frequency but not amplitude of mIPSCs was increased in female CA1 neurons (Figure 3 and Supplementary Figures 1A,B). These changes may involve some presynaptic changes given that Shank3 proteins are mainly localized at excitatory postsynaptic sites. Alternatively, the Shank3 protein with Q321R mutation might be localized at subcellular sites other than excitatory postsynaptic sites or even at inhibitory postsynaptic sites in CA1 pyramidal neurons, although our Shank3 antibodies were not good enough for immunohistochemical analyses at the electron microscopic level. Intriguingly, a similar increase in mIPSC frequency has been reported in *Shank3*^Δ9^ CA1 pyramidal neurons (Lee et al., 2015), although mIPSCs were not affected in Shank3^Δ4–9^ CA1 pyramidal neurons (Wang et al., 2011).

Our data indicate a decrease in neuronal excitability in male *Shank3*^Q321R/Q321R^ CA1 pyramidal neurons, a conclusion supported by the current-firing curve (Figure 3). However, there were no changes in the current-voltage relationship, input resistance, or Sag ratio. These results suggest potential changes in the threshold or properties of action potentials involving sodium or potassium channels, rather than changes in resting conductance involving HCN (hyperpolarization-activated cyclic nucleotide–gated) channels. Notably, a previous study has reported that cultured hippocampal neurons obtained from *Shank3* mice carrying heterozygous and homozygous deletion of exons 13–16 encoding the PDZ domain of Shank3 (Peca et al., 2011) display markedly increased neuronal excitability in association with increased input resistance and decreased hyperpolarization-activated currents (*I*_h_). Moreover, human neurons with a *SHANK3* exon 13 deletion display similar changes (Yi et al., 2016). This difference may be attributable to differences in the specific exons of the Shank3 gene affected: exon 8 in our study of the Q321R mutation and exons 13–16 in these previous studies. The former encompasses mainly ARR-containing Shank3 protein variants, whereas the latter encompasses almost all Shank3 protein variants. Although further details remain to be elucidated, our electrophysiology results suggest that, at minimum, the decreased neuronal excitability and increased mIPSC frequency in *Shank3*^Q321R/Q321R^ CA1 pyramidal neurons would substantially suppress the output functions of these neurons.

Behaviorally, heterozygous *Shank3*^+/Q321R^ mice show moderately enhanced social communication and self-grooming and moderate anxiolytic-like behaviors in the light-dark tests (although open-field center activity and EPM performance were normal), while showing normal levels of locomotor activity and three-chamber social interaction (Figure 4, 5). Homozygous *Shank3*^Q321R/Q321R^ mice show behaviors that are largely similar to those in heterozygous *Shank3*^+/Q321R^ mice, displaying moderately enhanced self-grooming, moderate anxiolytic-like behaviors in EPM and light-dark tests (although open-field center activity was normal), while showing normal levels of locomotor activity, social interaction (three-chamber and direct social interaction), social communication (courtship USV), and object–recognition and contextual fear memory (Figure 6–8; summarized in Table 1).

Notably, *Shank3*^Δ9^ mice, which carry a similar alteration in the ARR domain, show behaviors that are largely similar to those of *Shank3*^Q321R/Q321R^ mice, displaying normal levels of locomotor activity, anxiety-like behavior (open-field center time), social interaction and communication (three-chamber and pup USV), and object–recognition memory, although self-grooming was also normal in these mice (Lee et al., 2015). Behavioral impacts in other ARR-deletion–carrying mouse lines are more severe compared with those reported here, a difference that is likely attributable to larger deletions in the ARR domain of the Shank3 protein. Specifically, self-grooming is enhanced in *Shank3*^Δ4–9^ mice, similar to our mice, but these former mice additionally show altered social interaction and communication and impaired novel object–recognition memory (Bozdagi et al., 2010; Wang et al., 2011; Yang et al., 2012; Jaramillo et al., 2016; Dhamne et al., 2017). In addition, *Shank3*^Δ4–7^ mice show abnormal social novelty recognition, but normal self-grooming (Peca et al., 2011), again different from our mice.

Identifying synaptic mechanisms and neural circuits associated with the behavioral phenotypes in *Shank3*^Q321R^ mice will require additional investigation. Notably, however, while male *Shank3*^Q321R/Q321R^ mice show normal mEPSCs and mIPSCs in the hippocampus and anxiolytic-like behavior, enhanced self-grooming, and suppressed digging, female *Shank3*^Q321R/Q321R^ mice show increased mIPSC frequency in the hippocampus and normal anxiety-like behavior, self-grooming, and digging (Figure 3, 6, 7 and Supplementary Figures 1C–F). Therefore, the increased mIPSC frequency in female mice might represent a part of the compensatory changes that might have normalized the anxiety-like behaviors and repetitive behaviors in female *Shank3*^Q321R/Q321R^ mice.

In addition, the most strongly affected Shank3 protein variant in our mice was Shank3a, which is abundantly expressed in the striatum among many brain regions (Wang et al., 2014). In addition, the striatopallidal pathway has been shown to regulate self-grooming in *Shank3*^Δ13–16^ mice (Wang et al., 2017). In addition, the striatum has emerged as one of the key brain regions involved in the regulation of anxiety-like behaviors, in addition to the amygdala, bed nucleus of the stria terminalis, hippocampus, and ventromedial prefrontal cortex (Lago et al., 2017). Therefore, it is tempting to speculate that striatal dysfunctions might contribute to the self-grooming and anxiolytic-like behavior in *Shank3*^Q321R^ mice. Although male *Shank3*^Q321R/Q321R^ mice displayed normal levels of excitatory and inhibitory spontaneous synaptic transmission (mEPSCs and mIPSCs) in the dorsolateral striatum (Figure 3G,H), additional investigations might reveal some other striatal dysfunctions in synaptic transmission and plasticity. For instance, previous studies have reported decreased NMDA/AMPA ratio but normal mEPSCs and mIPSCs in the dorsal striatum of homozygous and heterozygous *Shank3*^Δ4–9^ mice (Jaramillo et al., 2016).

*Shank3*^Q321R/Q321R^ mice (males) display abnormal baseline EEG patterns, with a decreased delta band in the frontal lobe and increased alpha band in the parietal lobe (Figure 9). In addition, we found left-right asymmetry in the parietal, but not frontal, lobe of the *Shank3*^Q321R^ brain. Understanding the biological significances of these results would have to involve, for instance, additional analyses of sleep behaviors and rhythms in *Shank3*^Q321R/Q321R^ mice and acquisition of EEG- and sleep-related details from the individual carrying the Q321R mutation (Moessner et al., 2007). Nonetheless, our data are in line with the previous studies in ASD that have reported abnormalities in the power, coherence, and asymmetry of EEGs in the delta and alpha ranges in addition to other frequency ranges (Wang et al., 2013). Moreover, the individual carrying the *SHANK3* Q321R mutation was shown to display abnormal EEG patterns (Moessner et al., 2007), although it is unclear whether the abnormal EEGs were observed in similar frequency ranges. Notably, a previous study on *Shank3*^Δ13–16^ mice reported abnormally enhanced EEGs in the low gamma range (30–80 Hz), known to be associated with parvalbumin-positive GABAergic neurons (Sohal et al., 2009), but normal EEGs in other frequency ranges (Dhamne et al., 2017). This difference could again reflect differences in the exons deleted in the two mutant mouse lines, with *Shank3*^Δ13–16^ mice lacking a larger number of Shank3 splice variants.

Our *Shank3*^Q321R/Q321R^ mice (males) are more resistant to PTZ-induced seizures and do not display spontaneous behavioral seizures (Figure 10). These results are in line with the suppressed excitability of CA1 pyramidal neurons in these animals. Similar to our results, a previous study on *Shank3*^Δ13–16^ mice revealed a decrease in susceptibility to PTZ-induced seizures (Dhamne et al., 2017) and lack of spontaneous behavioral seizures (Peca et al., 2011). In addition, *Shank3*^Δ4–22^ mice show tonic hyperactivity in the cortico-striatal-thalamic axis, revealed by multi-site *in vivo* recordings, but do not show spontaneous behavioral seizures (Wang et al., 2016). In contrast, a transgenic mouse line carrying duplicated *Shank3* shows enhanced epileptiform spikes in the dentate gyrus and electrographic seizures (Han et al., 2013). These results collectively suggest that Shank3 is an important regulator of excitatory drive in the brain. Perhaps more importantly, the individual with the Q321R mutation was shown to display bilateral epileptiform discharges without seizures, in addition to abnormal EEG patterns (Moessner et al., 2007). Therefore, the EEG and seizure phenotypes of our *Shank3*^Q321R^ mice may serve as potential biomarkers for future studies.

## Conclusion

Our data suggest that the Shank3 Q321R mutation in mice has significant influences on Shank3 protein stability, hippocampal neuronal excitability, anxiety-like and repetitive behaviors, EEG, and seizure susceptibility.

## Ethics Statement

The mice were bred and maintained according to the Animal Research Requirements of KAIST, and all procedures were approved by the Committee of Animal Research at KAIST (2016-30).

## Author Contributions

JL generated the mice. Y-EY and TY performed the electrophysiological and behavioral experiments. Y-EY performed the immunoblot experiments. SL performed the immunohistochemistry and behavioral experiments. Y-EY, SL, and TY performed the EEG experiments. DK performed the structural modeling. H-MH, Y-CB, and EK wrote the manuscript.

## Conflict of Interest Statement

The authors declare that the research was conducted in the absence of any commercial or financial relationships that could be construed as a potential conflict of interest.

## Footnotes

1 http://folding.biofold.org/i-mutant/i-mutant2.0.html

## Supplementary Material

FIGURE S1 Female *Shank3*^Q321R/Q321R^ mice show increased mIPSC frequency in CA1 pyramidal neurons but normal anxiety-like and repetitive behaviors. **(A)** Normal mEPSCs in CA1 pyramidal neurons in the hippocampus of female *Shank3*^Q321R/Q321R^ mice (P21–27). The indicated values represent means ± SEM. *n* = 14 neurons from 4 mice (WT; female) and 15 neurons from 4 mice (Q321R), ns, not significant, Mann–Whitney *U*-test (for frequency) and Student’s *t*-test (for amplitude). **(B)** Increased frequency, but normal amplitude, of mIPSCs in CA1 pyramidal neurons in the hippocampus of female *Shank3*^Q321R/Q321R^ mice (P22–26). *n* = 12 neurons from 4 mice (WT) and 13 neurons from 4 mice (Q321R), ^∗^*P* < 0.05, ns, not significant, Student’s *t*-test. **(C)** Normal anxiety-like behavior in female *Shank3*^Q321R/Q321R^ mice (3–5 months) in the elevated plus-maze test, as shown by time spent in open arms of the maze. *n* = 9 mice (WT) and 12 mice (Q321R), ns, not significant, Mann–Whitney *U*-test. **(D)** Normal anxiety-like behavior in female *Shank3*^Q321R/Q321R^ mice (3–5 months) in the light-dark test, as shown by time spent in light chamber of the light-dark apparatus. *n* = 9 mice (WT) and 12 mice (Q321R), ns, not significant, Mann–Whitney *U*-test. **(E)** Normal self-grooming in female *Shank3*^Q321R/Q321R^ mice (3–5 months) in home cages with bedding (10 min), as shown by total self-grooming time. *n* = 9 mice (WT) and 11 mice (Q321R), ns, not significant, Welch’s *t*-test. **(F)** Normal digging in female *Shank3*^Q321R/Q321R^ mice (3–5 months) in home cages with bedding (10 min), as shown by total digging time. *n* = 9 mice (WT) and 11 mice (Q321R), ns, not significant, Student’s *t*-test.

FIGURE S2 *Shank3*^Q321R/Q321R^ mice show normal self-grooming in novel home cages without light, Laboras cages with light, and open-field arena with light. **(A)** Normal self-grooming in *Shank3*^Q321R/Q321R^ mice (4–5 months; male) in novel home cages without light (0 lux; 10 min). *n* = 16 mice (WT), 20 mice (Q321R), ns, not significant, Mann–Whitney *U*-test. **(B)** Normal self-grooming in *Shank3*^Q321R/Q321R^ mice (4–5 months; male) in Laboras cages (a novel environment) with light (∼50 lux; 10 min). *n* = 9 mice (WT), 8 mice (Q321R), ns, not significant, Mann–Whitney *U*-test. **(C)** Normal self-grooming in *Shank3*^Q321R/Q321R^ mice (2–3 months; male) in an open-field arena (a novel environment) with light (∼100 lux; 10 min). *n* = 13 mice (WT), 14 mice (Q321R), ns, not significant, Student’s *t*-test.

FIGURE S3 Abnormal EEG patterns in the left and right hemispheres in *Shank3*^Q321R/Q321R^ mice. **(A,B)** Altered left and right hemispheric EEG powers in the frontal and parietal lobes of the *Shank3*^Q321R/Q321R^ brain (3 months; male). Note that the frontal lobe displays decreased delta power in both left and right hemispheres, whereas the parietal lobe displays decreased delta power and increased theta and alpha powers only in the right hemisphere. *n* = 10 mice (WT) and 10 mice (Q321R) for frontal lobe and 9 mice (WT) and 10 mice (Q321R) for parietal lobe, ^∗^*P* < 0.05, ^∗∗^*P* < 0.01, ns, not significant, Student’s *t*-test, Mann–Whitney *U*-test, Welch’s *t*-test.

TABLE S1 Statistical details.

## References

[B1] Amal H. Barak B. Bhat V. Gong G. Joughin B. A. Wishnok J. S. (2018). Shank3 mutation in a mouse model of autism leads to changes in the S-nitroso-proteome and affects key proteins involved in vesicle release and synaptic function. *Mol. Psychiatry* [Epub ahead of print]. 29988084 10.1038/s41380-018-0113-6 PMC6614015

[B2] Amaral D. G. Schumann C. M. Nordahl C. W. (2008). Neuroanatomy of autism. *Trends Neurosci.* 31 137–145. 18258309 10.1016/j.tins.2007.12.005

[B3] Ankier S. I. (1974). New hot plate tests to quantify antinociceptive and narcotic antagonist activities. *Eur. J. Pharmacol.* 27 1–4. 10.1016/0014-2999(74)90195-2 4853341

[B4] Antunes M. Biala G. (2012). The novel object recognition memory: neurobiology, test procedure, and its modifications. *Cognit. Process* 13 93–110. 10.1007/s10339-011-0430-z 22160349 PMC3332351

[B5] Balaan C. Corley M. J. Eulalio T. Leite-Ahyo K. Pang A. P. S. Fang R. (2019). Juvenile Shank3b deficient mice present with behavioral phenotype relevant to autism spectrum disorder. *Behav. Brain Res.* 356 137–147. 10.1016/j.bbr.2018.08.005 30134148 PMC6247805

[B6] Berg E. L. Copping N. A. Rivera J. K. Pride M. C. Careaga M. Bauman M. D. (2018). Developmental social communication deficits in the Shank3 rat model of phelan-mcdermid syndrome and autism spectrum disorder. *Autism Res.* 11 587–601. 10.1002/aur.1925 29377611 PMC5903935

[B7] Bey A. L. Wang X. Yan H. Kim N. Passman R. L. Yang Y. (2018). Brain region-specific disruption of Shank3 in mice reveals a dissociation for cortical and striatal circuits in autism-related behaviors. *Transl. Psychiatry* 8:94. 29700290 10.1038/s41398-018-0142-6 PMC5919902

[B8] Boccuto L. Lauri M. Sarasua S. M. Skinner C. D. Buccella D. Dwivedi A. (2013). Prevalence of SHANK3 variants in patients with different subtypes of autism spectrum disorders. *Eur. J. Hum. Genet.* 21 310–316. 10.1038/ejhg.2012.175 22892527 PMC3573207

[B9] Bockers T. M. Mameza M. G. Kreutz M. R. Bockmann J. Weise C. Buck F. (2001). Synaptic scaffolding proteins in rat brain. Ankyrin repeats of the multidomain Shank protein family interact with the cytoskeletal protein alpha-fodrin. *J. Biol. Chem.* 276 40104–40112. .org/10.1074/jbc.m102454200 11509555 10.1074/jbc.M102454200

[B10] Bockmann J. Kreutz M. R. Gundelfinger E. D. Bockers T. M. (2002). ProSAP/Shank postsynaptic density proteins interact with insulin receptor tyrosine kinase substrate IRSp53. *J. Neurochem.* 83 1013–1017. 10.1046/j.1471-4159.2002.01204.x 12421375

[B11] Boeckers T. M. Bockmann J. Kreutz M. R. Gundelfinger E. D. (2002). ProSAP/Shank proteins – a family of higher order organizing molecules of the postsynaptic density with an emerging role in human neurological disease. *J. Neurochem.* 81 903–910. .org/10.1046/j.1471-4159.2002.00931.x 12065602 10.1046/j.1471-4159.2002.00931.x

[B12] Boeckers T. M. Kreutz M. R. Winter C. Zuschratter W. Smalla K. H. Sanmarti-Vila L. (1999). Proline-rich synapse-associated protein-1/cortactin binding protein 1 (ProSAP1/CortBP1) is a PDZ-domain protein highly enriched in the postsynaptic density. *J. Neurosci.* 19 6506–6518. 10.1523/jneurosci.19-15-06506.1999 10414979 PMC6782800

[B13] Bonaglia M. C. Giorda R. Beri S. De Agostini C. Novara F. Fichera M. (2011). Molecular mechanisms generating and stabilizing terminal 22q13 deletions in 44 subjects with Phelan/McDermid syndrome. *PLoS Genet.* 7:e1002173. 10.1371/journal.pgen.1002173 21779178 PMC3136441

[B14] Bonaglia M. C. Giorda R. Borgatti R. Felisari G. Gagliardi C. Selicorni A. (2001). Disruption of the ProSAP2 gene in a t(12;22)(q24.1;q13.3) is associated with the 22q13.3 deletion syndrome. *Am. J. Hum. Genet.* 69 261–268. 10.1086/321293 11431708 PMC1235301

[B15] Bourin M. Hascoet M. (2003). The mouse light/dark box test. *Eur. J. Pharmacol.* 463 55–65. 10.1016/s0014-2999(03)01274-3 12600702

[B16] Bozdagi O. Sakurai T. Papapetrou D. Wang X. Dickstein D. L. Takahashi N. (2010). Haploinsufficiency of the autism-associated Shank3 gene leads to deficits in synaptic function, social interaction, and social communication. *Mol. Autism* 1:15. 10.1186/2040-2392-1-15 21167025 PMC3019144

[B17] Capriotti E. Fariselli P. Casadio R. (2005). I-Mutant2.0: predicting stability changes upon mutation from the protein sequence or structure. *Nucleic Acids Res.* 33 W306–W310. 15980478 10.1093/nar/gki375 PMC1160136

[B18] Chung W. Choi S. Y. Lee E. Park H. Kang J. Park H. (2015). Social deficits in IRSp53 mutant mice improved by NMDAR and mGluR5 suppression. *Nat. Neurosci.* 18 435–443. 10.1038/nn.3927 25622145

[B19] Cochoy D. M. Kolevzon A. Kajiwara Y. Schoen M. Pascual-Lucas M. Lurie S. (2015). Phenotypic and functional analysis of SHANK3 stop mutations identified in individuals with ASD and/or ID. *Mol. Autism* 6:23. 10.1186/s13229-015-0020-5 PMC4455919 26045941

[B20] De Rubeis S. Siper P. M. Durkin A. Weissman J. Muratet F. Halpern D. (2018). Delineation of the genetic and clinical spectrum of Phelan-McDermid syndrome caused by SHANK3 point mutations. *Mol. Autism* 9:31. 29719671 10.1186/s13229-018-0205-9 PMC5921983

[B21] de Sena Cortabitarte A. Degenhardt F. Strohmaier J. Lang M. Weiss B. Roeth R. (2017). Investigation of SHANK3 in schizophrenia. *Am. J. Med. Genet. B Neuropsychiatr. Genet.* 174 390–398. .org/10.1002/ajmg.b.32528 28371232 10.1002/ajmg.b.32528

[B22] DeLano W. L. (2009). *PyMOL Molecular Viewer: Updates and Refinements.* Washington, DC: American Chemical Society, 238.

[B23] Dere E. Winkler D. Ritter C. Ronnenberg A. Poggi G. Patzig J. (2015). Gpm6b deficiency impairs sensorimotor gating and modulates the behavioral response to a 5-HT2A/C receptor agonist. *Behav. Brain Res.* 277 254–263. 10.1016/j.bbr.2014.04.021 24768641

[B24] Dhamne S. C. Silverman J. L. Super C. E. Lammers S. H. T. Hameed M. Q. Modi M. E. (2017). Replicable in vivo physiological and behavioral phenotypes of the Shank3B null mutant mouse model of autism. *Mol. Autism* 8:26. 28638591 10.1186/s13229-017-0142-z PMC5472997

[B25] Drapeau E. Riad M. Kajiwara Y. Buxbaum J. D. (2018). Behavioral phenotyping of an improved mouse model of phelan-mcdermid syndrome with a complete deletion of the Shank3 gene. *eNeuro* 5:ENEURO.46–ENEURO.18. 30302388 10.1523/ENEURO.0046-18.2018 PMC6175061

[B26] Du Y. Weed S. A. Xiong W. C. Marshall T. D. Parsons J. T. (1998). Identification of a novel cortactin SH3 domain-binding protein and its localization to growth cones of cultured neurons. *Mol. Cell Biol.* 18 5838–5851. 10.1128/mcb.18.10.5838 9742101 PMC109170

[B27] Duffney L. J. Zhong P. Wei J. Matas E. Cheng J. Qin L. (2015). Autism-like deficits in Shank3-deficient mice are rescued by targeting actin regulators. *Cell Rep.* 11 1400–1413. .org/10.1016/j.celrep.2015.04.064 26027926 10.1016/j.celrep.2015.04.064 PMC4464902

[B28] Durand C. M. Betancur C. Boeckers T. M. Bockmann J. Chaste P. Fauchereau F. (2007). Mutations in the gene encoding the synaptic scaffolding protein SHANK3 are associated with autism spectrum disorders. *Nat. Genet.* 39 25–27. 10.1038/ng1933 17173049 PMC2082049

[B29] Durand C. M. Perroy J. Loll F. Perrais D. Fagni L. Bourgeron T. (2012). SHANK3 mutations identified in autism lead to modification of dendritic spine morphology via an actin-dependent mechanism. *Mol. Psychiatry* 17 71–84. 10.1038/mp.2011.57 21606927 PMC3252613

[B30] Egnor S. R. Seagraves K. M. (2016). The contribution of ultrasonic vocalizations to mouse courtship. *Curr. Opin. Neurobiol.* 38 1–5. 10.1016/j.conb.2015.12.009 26789140

[B31] Ehlers M. D. (2003). Activity level controls postsynaptic composition and signaling via the ubiquitin-proteasome system. *Nat. Neurosci.* 6 231–242. 10.1038/nn1013 12577062

[B32] Engineer C. T. Rahebi K. C. Borland M. S. Buell E. P. Im K. W. Wilson L. G. (2018). Shank3-deficient rats exhibit degraded cortical responses to sound. *Autism Res.* 11 59–68. 10.1002/aur.1883 29052348 PMC5773383

[B33] Ferhat A. T. Halbedl S. Schmeisser M. J. Kas M. J. Bourgeron T. Ey E. (2017). Behavioural phenotypes and neural circuit dysfunctions in mouse models of autism spectrum disorder. *Adv. Anat. Embryol. Cell Biol.* 224 85–101. 10.1007/978-3-319-52498-6_5 28551752

[B34] Figura M. G. Coppola A. Bottitta M. Calabrese G. Grillo L. Luciano D. (2014). Seizures and EEG pattern in the 22q13.3 deletion syndrome: clinical report of six Italian cases. *Seizure* 23 774–779. .org/10.1016/j.seizure.2014.06.008 25027555 10.1016/j.seizure.2014.06.008

[B35] Fiser A. Do R. K. Sali A. (2000). Modeling of loops in protein structures. *Protein Sci.* 9 1753–1773. 10.1110/ps.9.9.1753 11045621 PMC2144714

[B36] Fourie C. Vyas Y. Lee K. Jung Y. Garner C. C. Montgomery J. M. (2018). Dietary zinc supplementation prevents autism related behaviors and striatal synaptic dysfunction in Shank3 exon 13-16 mutant mice. *Front. Cell Neurosci.* 12:374. 10.3389/fncel.2018.00374 30405356 PMC6204368

[B37] Gauthier J. Champagne N. Lafreniere R. G. Xiong L. Spiegelman D. Brustein E. (2010). De novo mutations in the gene encoding the synaptic scaffolding protein SHANK3 in patients ascertained for schizophrenia. *Proc. Natl. Acad. Sci. U.S.A.* 107 7863–7868. .org/10.1073/pnas.0906232107 20385823 10.1073/pnas.0906232107 PMC2867875

[B38] Gould T. D. Dao D. T. Kovacsics C. E. (2009). “The open field test,” in *Mood and Anxiety Related Phenotypes in Mice*, ed. Gould T. D. (Totowa, NJ: Humana Press).

[B39] Grabrucker A. M. Schmeisser M. J. Schoen M. Boeckers T. M. (2011). Postsynaptic ProSAP/Shank scaffolds in the cross-hair of synaptopathies. *Trends Cell Biol.* 21 594–603. .org/10.1016/j.tcb.2011.07.003 21840719 10.1016/j.tcb.2011.07.003

[B40] Guilmatre A. Huguet G. Delorme R. Bourgeron T. (2014). The emerging role of SHANK genes in neuropsychiatric disorders. *Dev. Neurobiol.* 74 113–122. 10.1002/dneu.22128 24124131

[B41] Hamdan F. F. Gauthier J. Araki Y. Lin D. T. Yoshizawa Y. Higashi K. (2011). Excess of de novo deleterious mutations in genes associated with glutamatergic systems in nonsyndromic intellectual disability. *Am. J. Hum. Genet.* 88 306–316. 10.1016/j.ajhg.2011.02.001 21376300 PMC3059427

[B42] Han K. Holder J. L. Jr. Schaaf C. P. Lu H. Chen H. (2013). SHANK3 overexpression causes manic-like behaviour with unique pharmacogenetic properties. *Nature* 503 72–77. 10.1038/nature12630 24153177 PMC3923348

[B43] Harony-Nicolas H. De Rubeis S. Kolevzon A. Buxbaum J. D. (2015). Phelan McDermid syndrome: from genetic discoveries to animal models and treatment. *J. Child Neurol.* 30 1861–1870. .org/10.1177/0883073815600872 26350728 10.1177/0883073815600872 PMC5321557

[B44] Harony-Nicolas H. Kay M. Hoffmann J. D. Klein M. E. Bozdagi-Gunal O. Riad M. (2017). Oxytocin improves behavioral and electrophysiological deficits in a novel Shank3-deficient rat. *Elife* 6:e18904. 28139198 10.7554/eLife.18904 PMC5283828

[B45] Heise C. Preuss J. M. Schroeder J. C. Battaglia C. R. Kolibius J. Schmid R. (2018). Heterogeneity of cell surface glutamate and GABA receptor expression in shank and CNTN4 autism mouse models. *Front. Mol. Neurosci.* 11:212. 10.3389/fnmol.2018.00212 29970989 PMC6018460

[B46] Heise C. Schroeder J. C. Schoen M. Halbedl S. Reim D. Woelfle S. (2016). Selective localization of shanks to VGLUT1-positive excitatory synapses in the mouse hippocampus. *Front. Cell. Neurosci.* 10:106. 10.3389/fncel.2016.00106 27199660 PMC4844616

[B47] Holder J. L. Jr. Quach M. M. (2016). The spectrum of epilepsy and electroencephalographic abnormalities due to SHANK3 loss-of-function mutations. *Epilepsia* 57 1651–1659. 10.1111/epi.13506 27554343 PMC5547884

[B48] Hrdinka M. Gyrd-Hansen M. (2017). The met1-linked ubiquitin machinery: emerging themes of (De)regulation. *Mol. Cell* 68 265–280. 10.1016/j.molcel.2017.09.001 29053955

[B49] Ikeda F. Deribe Y. L. Skanland S. S. Stieglitz B. Grabbe C. Franz-Wachtel M. (2011). SHARPIN forms a linear ubiquitin ligase complex regulating NF-kappaB activity and apoptosis. *Nature* 471 637–641. .org/10.1038/nature09814 21455181 10.1038/nature09814 PMC3085511

[B50] Ikeda K. Araki K. Takayama C. Inoue Y. Yagi T. Aizawa S. (1995). Reduced spontaneous activity of mice defective in the epsilon 4 subunit of the NMDA receptor channel. *Brain Res. Mol. Brain Res.* 33 61–71. 10.1016/0169-328x(95)00107-4 8774946

[B51] Iwai K. Fujita H. Sasaki Y. (2014). Linear ubiquitin chains: NF-kappaB signalling, cell death and beyond. *Nat. Rev. Mol. Cell Biol.* 15 503–508. 10.1038/nrm3836 25027653

[B52] Jaramillo T. C. Speed H. E. Xuan Z. Reimers J. M. Escamilla C. O. Weaver T. P. (2017). Novel Shank3 mutant exhibits behaviors with face validity for autism and altered striatal and hippocampal function. *Autism Res.* 10 42–65. 10.1002/aur.1664 27492494 PMC5274551

[B53] Jaramillo T. C. Speed H. E. Xuan Z. Reimers J. M. Liu S. Powell C. M. (2016). Altered striatal synaptic function and abnormal behaviour in Shank3 Exon4-9 deletion mouse model of autism. *Autism Res.* 9 350–375. 10.1002/aur.1529 26559786 PMC4857590

[B54] Jiang Y. H. Ehlers M. D. (2013). Modeling autism by SHANK gene mutations in mice. *Neuron* 78 8–27. 10.1016/j.neuron.2013.03.016 23583105 PMC3659167

[B55] Jin C. Kang H. Ryu J. R. Kim S. Zhang Y. Lee Y. (2018). Integrative brain transcriptome analysis reveals region-specific and broad molecular changes in Shank3-overexpressing mice. *Front. Mol. Neurosci.* 11:250. 10.3389/fnmol.2018.00250 30233305 PMC6127286

[B56] Kerrisk Campbell M. Sheng M. (2018). USP8 deubiquitinates SHANK3 to control synapse density and SHANK3 activity-dependent protein levels. *J. Neurosci.* 38 5289–5301. .org/10.1523/jneurosci.3305-17.2018 29735556 10.1523/JNEUROSCI.3305-17.2018 PMC6596000

[B57] Kouser M. Speed H. E. Dewey C. M. Reimers J. M. Widman A. J. Gupta N. (2013). Loss of predominant Shank3 isoforms results in hippocampus-dependent impairments in behavior and synaptic transmission. *J. Neurosci.* 33 18448–18468. 10.1523/jneurosci.3017-13.2013 24259569 PMC3834052

[B58] Lago T. Davis A. Grillon C. Ernst M. (2017). Striatum on the anxiety map: small detours into adolescence. *Brain Res.* 1654 177–184. 10.1016/j.brainres.2016.06.006 27276526 PMC5140771

[B59] Leblond C. S. Heinrich J. Delorme R. Proepper C. Betancur C. Huguet G. (2012). Genetic and functional analyses of SHANK2 mutations suggest a multiple hit model of autism spectrum disorders. *PLoS Genet.* 8:e1002521. 10.1371/journal.pgen.1002521 22346768 PMC3276563

[B60] Leblond C. S. Nava C. Polge A. Gauthier J. Huguet G. Lumbroso S. (2014). Meta-analysis of SHANK mutations in autism spectrum disorders: a gradient of severity in cognitive impairments. *PLoS Genet.* 10:e1004580. 10.1371/journal.pgen.1004580 25188300 PMC4154644

[B61] Lee J. Chung C. Ha S. Lee D. Kim D. Y. Kim H. (2015). Shank3-mutant mice lacking exon 9 show altered excitation/inhibition balance, enhanced rearing, and spatial memory deficit. *Front. Cell Neurosci.* 9:94. 10.3389/fncel.2015.00094 25852484 PMC4365696

[B62] Lee S. Ahmed T. Lee S. Kim H. Choi S. Kim D. S. (2012). Bidirectional modulation of fear extinction by mediodorsal thalamic firing in mice. *Nat. Neurosci.* 15 308–314. 10.1038/nn.2999 22197828

[B63] Lee S. Lee E. Kim R. Kim J. Lee S. Park H. (2018). Shank2 deletion in parvalbumin neurons leads to moderate hyperactivity, enhanced self-grooming and suppressed seizure susceptibility in mice. *Front. Mol. Neurosci.* 11:209. 10.3389/fnmol.2018.00209 29970987 PMC6018407

[B64] Lee Y. Kim S. G. Lee B. Zhang Y. Kim Y. Kim S. (2017). Striatal transcriptome and interactome analysis of Shank3-overexpressing mice reveals the connectivity between Shank3 and mTORC1 signaling. *Front. Mol. Neurosci.* 10:201. 10.3389/fnmol.2017.00201 28701918 PMC5487420

[B65] Lilja J. Zacharchenko T. Georgiadou M. Jacquemet G. De Franceschi N. Peuhu E. (2017). SHANK proteins limit integrin activation by directly interacting with Rap1 and R-Ras. *Nat. Cell Biol.* 19 292–305. 10.1038/ncb3487 28263956 PMC5386136

[B66] Lim S. Naisbitt S. Yoon J. Hwang J. I. Suh P. G. Sheng M. (1999). Characterization of the shank family of synaptic proteins. Multiple genes, alternative splicing, and differential expression in brain and development. *J. Biol. Chem.* 274 29510–29518. .org/10.1074/jbc.274.41.29510 10506216 10.1074/jbc.274.41.29510

[B67] Lim S. Sala C. Yoon J. Park S. Kuroda S. Sheng M. (2001). Sharpin, a novel postsynaptic density protein that directly interacts with the shank family of proteins. *Mol. Cell. Neurosci.* 17 385–397. 10.1006/mcne.2000.0940 11178875

[B68] Ma K. Qin L. Matas E. Duffney L. J. Liu A. Yan Z. (2018). Histone deacetylase inhibitor MS-275 restores social and synaptic function in a Shank3-deficient mouse model of autism. *Neuropsychopharmacology* 43 1779–1788. 10.1038/s41386-018-0073-1 29760409 PMC6006368

[B69] Maggio J. C. Whitney G. (1985). Ultrasonic vocalizing by adult female mice (Mus musculus). *J. Comp. Psychol.* 99 420–436. .org/10.1037//0735-7036.99.4.420 4075780

[B70] Mameza M. G. Dvoretskova E. Bamann M. Honck H. H. Guler T. Boeckers T. M. (2013). SHANK3 gene mutations associated with autism facilitate ligand binding to the Shank3 ankyrin repeat region. *J. Biol. Chem.* 288 26697–26708. 10.1074/jbc.m112.424747 23897824 PMC3772216

[B71] Maunakea A. K. Nagarajan R. P. Bilenky M. Ballinger T. J. D’souza C. Fouse S. D. (2010). Conserved role of intragenic DNA methylation in regulating alternative promoters. *Nature* 466 253–257. 10.1038/nature09165 20613842 PMC3998662

[B72] Mei Y. Monteiro P. Zhou Y. Kim J. A. Gao X. Fu Z. (2016). Adult restoration of Shank3 expression rescues selective autistic-like phenotypes. *Nature* 530 481–484. 10.1038/nature16971 26886798 PMC4898763

[B73] Milad M. R. Quirk G. J. (2002). Neurons in medial prefrontal cortex signal memory for fear extinction. *Nature* 420 70–74. 10.1038/nature01138 12422216

[B74] Moessner R. Marshall C. R. Sutcliffe J. S. Skaug J. Pinto D. Vincent J. (2007). Contribution of SHANK3 mutations to autism spectrum disorder. *Am. J. Hum. Genet.* 81 1289–1297. 10.1086/522590 17999366 PMC2276348

[B75] Monteiro P. Feng G. (2017). SHANK proteins: roles at the synapse and in autism spectrum disorder. *Nat. Rev. Neurosci.* 18 147–157. 10.1038/nrn.2016.183 28179641

[B76] Mossa A. Giona F. Pagano J. Sala C. Verpelli C. (2017). SHANK genes in autism: defining therapeutic targets. *Prog. Neuropsychopharmacol. Biol. Psychiatry* 84 416–423. 10.1016/j.pnpbp.2017.11.019 29175319

[B77] Moy S. S. Nadler J. J. Perez A. Barbaro R. P. Johns J. M. Magnuson T. R. (2004). Sociability and preference for social novelty in five inbred strains: an approach to assess autistic-like behavior in mice. *Genes Brain Behav.* 3 287–302. 10.1111/j.1601-1848.2004.00076.x 15344922

[B78] Nadler J. J. Moy S. S. Dold G. Trang D. Simmons N. Perez A. (2004). Automated apparatus for quantitation of social approach behaviors in mice. *Genes Brain Behav.* 3 303–314. 10.1111/j.1601-183x.2004.00071.x 15344923

[B79] Naisbitt S. Kim E. Tu J. C. Xiao B. Sala C. Valtschanoff J. (1999). Shank, a novel family of postsynaptic density proteins that binds to the NMDA receptor/PSD-95/GKAP complex and cortactin. *Neuron* 23 569–582. 10.1016/s0896-6273(00)80809-0 10433268

[B80] Naydenov A. V. Horne E. A. Cheah C. S. Swinney K. Hsu K. L. Cao J. K. (2014). ABHD6 blockade exerts antiepileptic activity in PTZ-induced seizures and in spontaneous seizures in R6/2 mice. *Neuron* 83 361–371. 10.1016/j.neuron.2014.06.030 25033180 PMC4136499

[B81] Nemirovsky S. I. Cordoba M. Zaiat J. J. Completa S. P. Vega P. A. Gonzalez-Moron D. (2015). Whole genome sequencing reveals a de novo SHANK3 mutation in familial autism spectrum disorder. *PLoS One* 10:e0116358. 10.1371/journal.pone.0116358 25646853 PMC4315573

[B82] Peca J. Feliciano C. Ting J. T. Wang W. Wells M. F. Venkatraman T. N. (2011). Shank3 mutant mice display autistic-like behaviours and striatal dysfunction. *Nature* 472 437–442. 10.1038/nature09965 21423165 PMC3090611

[B83] Peixoto R. T. Wang W. Croney D. M. Kozorovitskiy Y. Sabatini B. L. (2016). Early hyperactivity and precocious maturation of corticostriatal circuits in Shank3B(-/-) mice. *Nat. Neurosci.* 19 716–724. .org/10.1038/nn.4260 26928064 10.1038/nn.4260 PMC4846490

[B84] Pellow S. Chopin P. File S. E. Briley M. (1985). Validation of open:closed arm entries in an elevated plus-maze as a measure of anxiety in the rat. *J. Neurosci. Methods* 14 149–167. .org/10.1016/0165-0270(85)90031-7 2864480 10.1016/0165-0270(85)90031-7

[B85] Phillips R. G. LeDoux J. E. (1992). Differential contribution of amygdala and hippocampus to cued and contextual fear conditioning. *Behav. Neurosci.* 106 274–285. 10.1037//0735-7044.106.2.274 1590953

[B86] Portfors C. V. (2007). Types and functions of ultrasonic vocalizations in laboratory rats and mice. *J. Am. Assoc. Lab. Anim. Sci.* 46 28–34. 17203913

[B87] Qin L. Ma K. Wang Z. J. Hu Z. Matas E. Wei J. (2018). Social deficits in Shank3-deficient mouse models of autism are rescued by histone deacetylase (HDAC) inhibition. *Nat. Neurosci.* 21 564–575. .org/10.1038/s41593-018-0110-8 29531362 10.1038/s41593-018-0110-8 PMC5876144

[B88] Quinn L. P. Stean T. O. Chapman H. Brown M. Vidgeon-Hart M. Upton N. (2006). Further validation of LABORAS using various dopaminergic manipulations in mice including MPTP-induced nigro-striatal degeneration. *J. Neurosci. Methods* 156 218–227. .org/10.1016/j.jneumeth.2006.03.013 16626808 10.1016/j.jneumeth.2006.03.013

[B89] Quinn L. P. Stean T. O. Trail B. Duxon M. S. Stratton S. C. Billinton A. (2003). LABORAS: initial pharmacological validation of a system allowing continuous monitoring of laboratory rodent behaviour. *J. Neurosci. Methods* 130 83–92. 10.1016/s0165-0270(03)00227-9 14583407

[B90] Rendall A. R. Perrino P. A. Buscarello A. N. Fitch R. H. (2019). Shank3B mutant mice display pitch discrimination enhancements and learning deficits. *Int. J. Dev. Neurosci.* 72 13–21. 10.1016/j.ijdevneu.2018.10.003 30385192

[B91] Rubenstein J. L. Merzenich M. M. (2003). Model of autism: increased ratio of excitation/inhibition in key neural systems. *Genes Brain Behav.* 2 255–267. 10.1034/j.1601-183x.2003.00037.x 14606691 PMC6748642

[B92] Sala C. Vicidomini C. Bigi I. Mossa A. Verpelli C. (2015). Shank synaptic scaffold proteins: keys to understanding the pathogenesis of autism and other synaptic disorders. *J. Neurochem.* 135 849–858. .org/10.1111/jnc.13232 26338675 10.1111/jnc.13232

[B93] Schmeisser M. J. Ey E. Wegener S. Bockmann J. Stempel A. V. Kuebler A. (2012). Autistic-like behaviours and hyperactivity in mice lacking ProSAP1/Shank2. *Nature* 486 256–260. 10.1038/nature11015 22699619

[B94] Sheng M. Hoogenraad C. C. (2007). The postsynaptic architecture of excitatory synapses: a more quantitative view. *Annu. Rev. Biochem.* 76 823–847. 10.1146/annurev.biochem.76.060805.160029 17243894

[B95] Sheng M. Kim E. (2000). The Shank family of scaffold proteins. *J. Cell Sci.* 113(Pt 11), 1851–1856. 10806096 10.1242/jcs.113.11.1851

[B96] Sheng M. Kim E. (2011). The postsynaptic organization of synapses. *Cold Spring Harb. Perspect. Biol.* 3:a005678. 10.1101/cshperspect.a005678 22046028 PMC3225953

[B97] Sheng M. Sala C. (2001). PDZ domains and the organization of supramolecular complexes. *Annu. Rev. Neurosci.* 24 1–29. 10.1146/annurev.neuro.24.1.1 11283303

[B98] Silverman J. L. Yang M. Lord C. Crawley J. N. (2010). Behavioural phenotyping assays for mouse models of autism. *Nat. Rev. Neurosci.* 11 490–502. 10.1038/nrn2851 20559336 PMC3087436

[B99] Sohal V. S. Zhang F. Yizhar O. Deisseroth K. (2009). Parvalbumin neurons and gamma rhythms enhance cortical circuit performance. *Nature* 459 698–702. 10.1038/nature07991 19396159 PMC3969859

[B100] Soorya L. Kolevzon A. Zweifach J. Lim T. Dobry Y. Schwartz L. (2013). Prospective investigation of autism and genotype-phenotype correlations in 22q13 deletion syndrome and SHANK3 deficiency. *Mol. Autism* 4 18. 10.1186/2040-2392-4-18 23758760 PMC3707861

[B101] Speed H. E. Kouser M. Xuan Z. Reimers J. M. Ochoa C. F. Gupta N. (2015). Autism-associated insertion mutation (InsG) of Shank3 Exon 21 causes impaired synaptic transmission and behavioral deficits. *J. Neurosci.* 35 9648–9665. 10.1523/jneurosci.3125-14.2015 26134648 PMC4571502

[B102] Stefanko D. P. Barrett R. M. Ly A. R. Reolon G. K. Wood M. A. (2009). Modulation of long-term memory for object recognition via HDAC inhibition. *Proc. Natl. Acad. Sci. U.S.A.* 106 9447–9452. 10.1073/pnas.0903964106 19470462 PMC2695069

[B103] Tan Q. Zoghbi H. Y. (2018). Mouse models as a tool for discovering new neurological diseases. *Neurobiol. Learn Mem.* [Epub ahead of print]. 30030131 10.1016/j.nlm.2018.07.006

[B104] Tokunaga F. Nakagawa T. Nakahara M. Saeki Y. Taniguchi M. Sakata S. (2011). SHARPIN is a component of the NF-kappaB-activating linear ubiquitin chain assembly complex. *Nature* 471 633–636. 10.1038/nature09815 21455180

[B105] Tu J. C. Xiao B. Naisbitt S. Yuan J. P. Petralia R. S. Brakeman P. (1999). Coupling of mGluR/Homer and PSD-95 complexes by the Shank family of postsynaptic density proteins. *Neuron* 23 583–592. 10.1016/s0896-6273(00)80810-7 10433269

[B106] Van de Weerd H. A. Bulthuis R. J. Bergman A. F. Schlingmann F. Tolboom J. Van Loo P. L. (2001). Validation of a new system for the automatic registration of behaviour in mice and rats. *Behav. Processes* 53 11–20. 10.1016/s0376-6357(00)00135-2 11254988

[B107] Vicidomini C. Ponzoni L. Lim D. Schmeisser M. J. Reim D. Morello N. (2017). Pharmacological enhancement of mGlu5 receptors rescues behavioral deficits in SHANK3 knock-out mice. *Mol. Psychiatry* 22 689–702. 10.1038/mp.2016.30 27021819 PMC5014121

[B108] Vogel-Ciernia A. Wood M. A. (2014). Examining object location and object recognition memory in mice. *Curr. Protoc. Neurosci.* 69 8 31–17. 25297693 10.1002/0471142301.ns0831s69 PMC4219523

[B109] Waga C. Asano H. Sanagi T. Suzuki E. Nakamura Y. Tsuchiya A. (2014). Identification of two novel Shank3 transcripts in the developing mouse neocortex. *J. Neurochem.* 128 280–293. 10.1111/jnc.12505 24164323

[B110] Wang J. Barstein J. Ethridge L. E. Mosconi M. W. Takarae Y. Sweeney J. A. (2013). Resting state EEG abnormalities in autism spectrum disorders. *J. Neurodev. Disord.* 5:24. 10.1186/1866-1955-5-24 PMC3847481 24040879

[B111] Wang L. Pang K. Han K. Adamski C. J. Wang W. He L. (2019). An autism-linked missense mutation in SHANK3 reveals the modularity of Shank3 function. *Mol. Psychiatry* [Epub ahead of print]. 30610205 10.1038/s41380-018-0324-x PMC6609509

[B112] Wang W. Li C. Chen Q. Van Der Goes M. S. Hawrot J. Yao A. Y. (2017). Striatopallidal dysfunction underlies repetitive behavior in Shank3-deficient model of autism. *J. Clin. Investig.* 127 1978–1990. .org/10.1172/jci87997 28414301 10.1172/JCI87997 PMC5409790

[B113] Wang X. Bey A. L. Katz B. M. Badea A. Kim N. David L. K. (2016). Altered mGluR5-Homer scaffolds and corticostriatal connectivity in a Shank3 complete knockout model of autism. *Nat. Commun.* 7:11459. 27161151 10.1038/ncomms11459 PMC4866051

[B114] Wang X. Mccoy P. A. Rodriguiz R. M. Pan Y. Je H. S. Roberts A. C. (2011). Synaptic dysfunction and abnormal behaviors in mice lacking major isoforms of Shank3. *Hum. Mol. Genet.* 20 3093–3108. .org/10.1093/hmg/ddr212 21558424 10.1093/hmg/ddr212 PMC3131048

[B115] Wang X. Xu Q. Bey A. L. Lee Y. Jiang Y. H. (2014). Transcriptional and functional complexity of Shank3 provides a molecular framework to understand the phenotypic heterogeneity of SHANK3 causing autism and Shank3 mutant mice. *Mol. Autism* 5:30. .org/10.1186/2040-2392-5-30 25071925 10.1186/2040-2392-5-30 PMC4113141

[B116] Wilson H. L. Wong A. C. Shaw S. R. Tse W. Y. Stapleton G. A. Phelan M. C. (2003). Molecular characterisation of the 22q13 deletion syndrome supports the role of haploinsufficiency of SHANK3/PROSAP2 in the major neurological symptoms. *J. Med. Genet.* 40 575–584. .org/10.1136/jmg.40.8.575 12920066 10.1136/jmg.40.8.575 PMC1735560

[B117] Yang M. Bozdagi O. Scattoni M. L. Wohr M. Roullet F. I. Katz A. M. (2012). Reduced excitatory neurotransmission and mild autism-relevant phenotypes in adolescent Shank3 null mutant mice. *J. Neurosci.* 32 6525–6541. 10.1523/jneurosci.6107-11.2012 22573675 PMC3362928

[B118] Yao I. Hata Y. Hirao K. Deguchi M. Ide N. Takeuchi M. (1999). Synamon, a novel neuronal protein interacting with synapse-associated protein 90/Postsynaptic density-95-associated protein. *J. Biol. Chem.* 274 27463–27466. 10.1074/jbc.274.39.27463 10488079

[B119] Yi F. Danko T. Botelho S. C. Patzke C. Pak C. Wernig M. (2016). Autism-associated SHANK3 haploinsufficiency causes Ih channelopathy in human neurons. *Science* 352:aaf2669. 10.1126/science.aaf2669 26966193 PMC4901875

[B120] Yoo T. Cho H. Lee J. Park H. Yoo Y. E. Yang E. (2018). GABA neuronal deletion of Shank3 exons 14-16 in mice suppresses striatal excitatory synaptic input and induces social and locomotor abnormalities. *Front. Cell Neurosci.* 12:341. 10.3389/fncel.2018.00341 30356810 PMC6189516

[B121] Zhou Y. Kaiser T. Monteiro P. Zhang X. Van Der Goes M. S. Wang D. (2016). Mice with Shank3 mutations associated with ASD and schizophrenia display both shared and distinct defects. *Neuron* 89 147–162. 10.1016/j.neuron.2015.11.023 26687841 PMC4754122

[B122] Zhu L. Wang X. Li X. L. Towers A. Cao X. Wang P. (2014). Epigenetic dysregulation of SHANK3 in brain tissues from individuals with autism spectrum disorders. *Hum. Mol. Genet.* 23 1563–1578. 10.1093/hmg/ddt547 24186872 PMC3929093

[B123] Zhu M. Idikuda V. K. Wang J. Wei F. Kumar V. Shah N. (2018). Shank3-deficient thalamocortical neurons show HCN channelopathy and alterations in intrinsic electrical properties. *J. Physiol.* 596 1259–1276. 10.1113/jp275147 29327340 PMC5878232

