# Supplementary figures and images for: Shank3 Mice Carrying the Human Q321R Mutation Display Enhanced Self-Grooming, Abnormal Electroencephalogram Patterns, and Suppressed Neuronal Excitability and Seizure Susceptibility
Source: Front Mol Neurosci. 2019 Jun 18;12:155. doi: 10.3389/fnmol.2019.00155 (PMC6591539; doi:10.3389/fnmol.2019.00155)

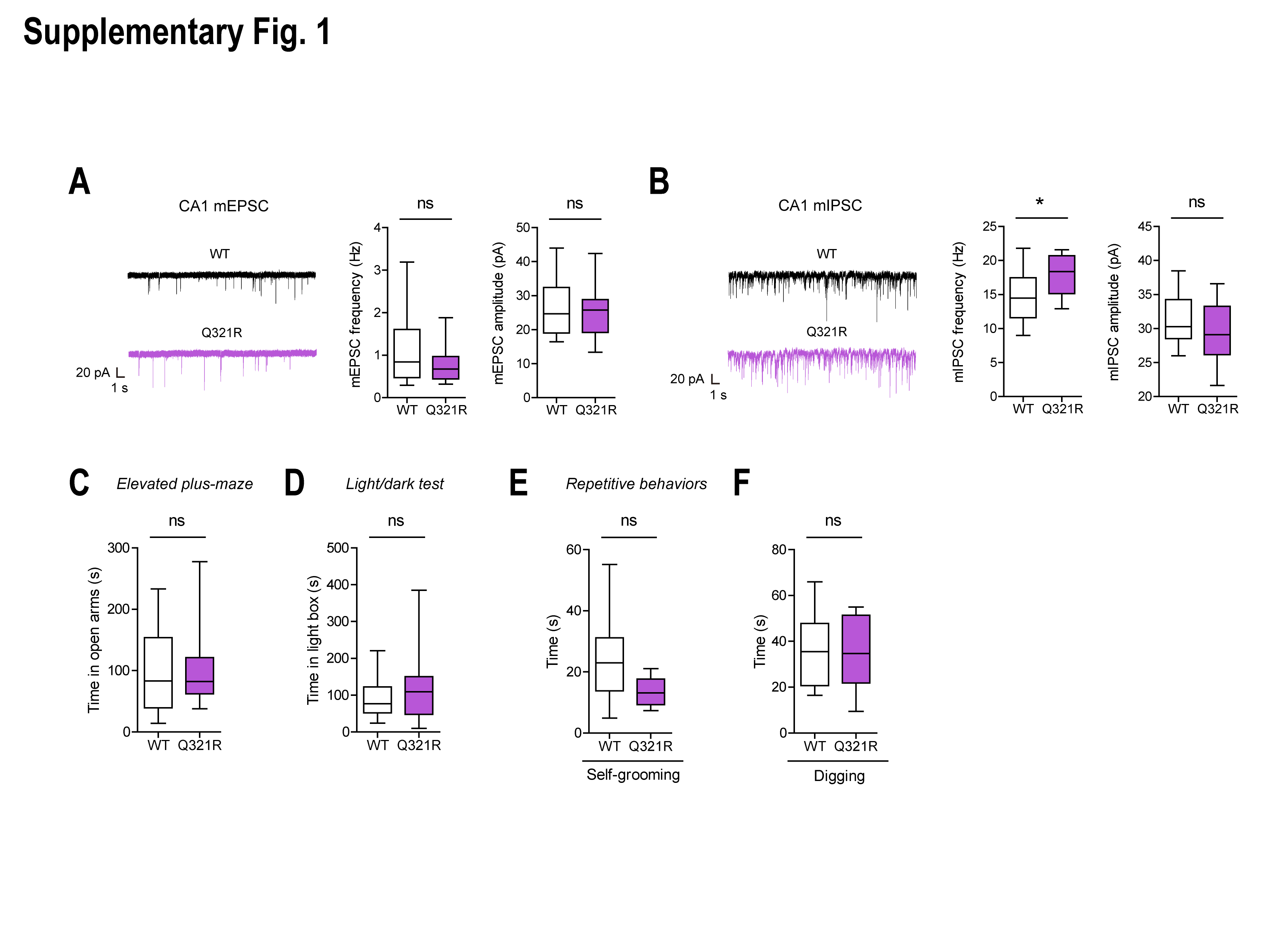

Supplement: FIGURE S1 — Female Shank3Q321R/Q321R mice show increased mIPSC frequency in CA1 pyramidal neurons but normal anxiety-like and repetitive behaviors. (A) Normal mEPSCs in CA1 pyramidal neurons in the hippocampus of female Shank3Q321R/Q321R mice (P21–27). The indicated values represent means ± SEM. n = 14 neurons from 4 mice (WT; female) and 15 neurons from 4 mice (Q321R), ns, not significant, Mann–Whitney U-test (for frequency) and Student’s t-test (for amplitude). (B) Increased frequency, but normal amplitude, of mIPSCs in CA1 pyramidal neurons in the hippocampus of female Shank3Q321R/Q321R mice (P22–26). n = 12 neurons from 4 mice (WT) and 13 neurons from 4 mice (Q321R), ∗P < 0.05, ns, not significant, Student’s t-test. (C) Normal anxiety-like behavior in female Shank3Q321R/Q321R mice (3–5 months) in the elevated plus-maze test, as shown by time spent in open arms of the maze. n = 9 mice (WT) and 12 mice (Q321R), ns, not significant, Mann–Whitney U-test. (D) Normal anxiety-like behavior in female Shank3Q321R/Q321R mice (3–5 months) in the light-dark test, as shown by time spent in light chamber of the light-dark apparatus. n = 9 mice (WT) and 12 mice (Q321R), ns, not significant, Mann–Whitney U-test. (E) Normal self-grooming in female Shank3Q321R/Q321R mice (3–5 months) in home cages with bedding (10 min), as shown by total self-grooming time. n = 9 mice (WT) and 11 mice (Q321R), ns, not significant, Welch’s t-test. (F) Normal digging in female Shank3Q321R/Q321R mice (3–5 months) in home cages with bedding (10 min), as shown by total digging time. n = 9 mice (WT) and 11 mice (Q321R), ns, not significant, Student’s t-test. [file Image_1.TIF]

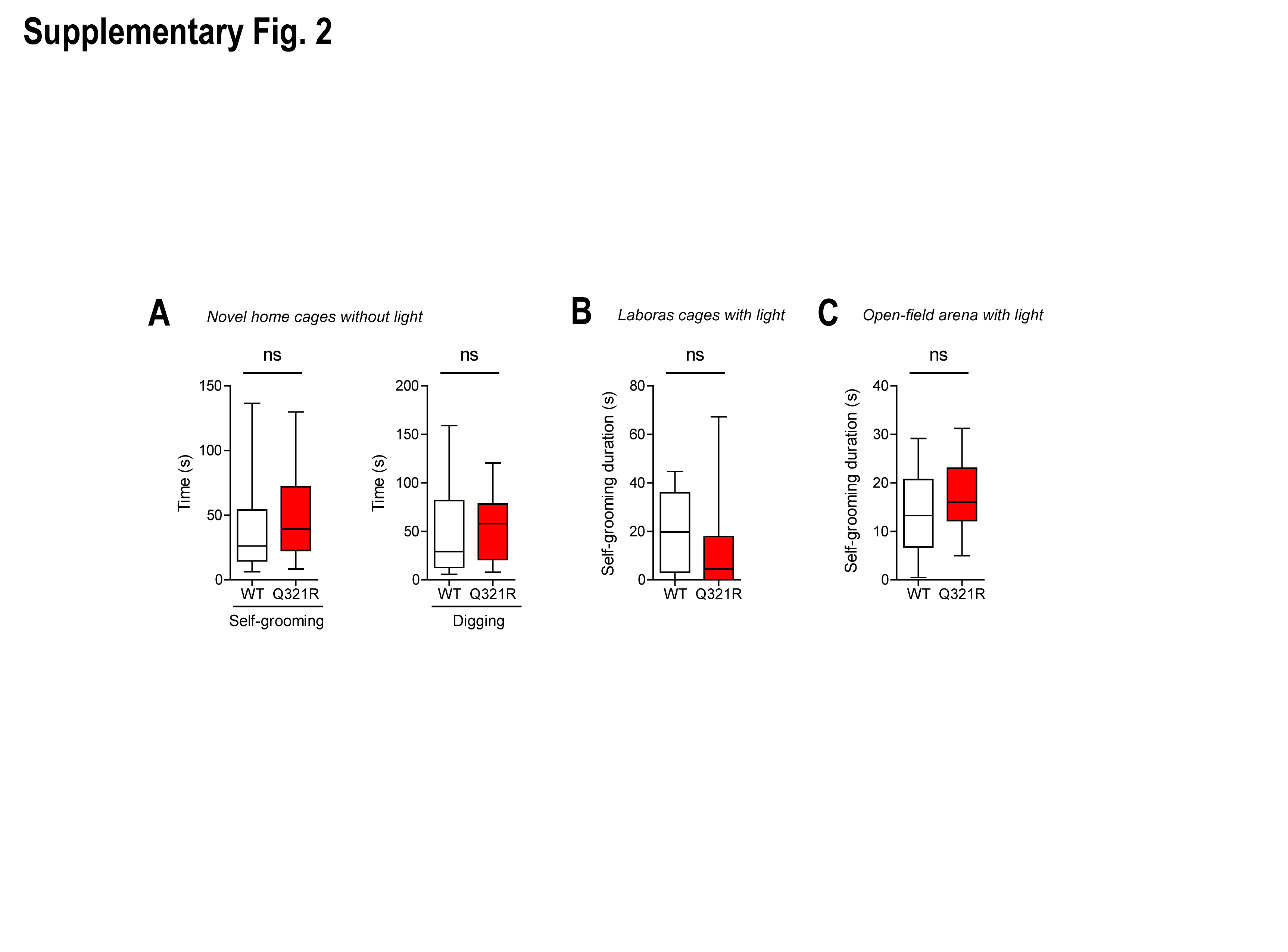

Supplement: FIGURE S2 — Shank3Q321R/Q321R mice show normal self-grooming in novel home cages without light, Laboras cages with light, and open-field arena with light. (A) Normal self-grooming in Shank3Q321R/Q321R mice (4–5 months; male) in novel home cages without light (0 lux; 10 min). n = 16 mice (WT), 20 mice (Q321R), ns, not significant, Mann–Whitney U-test. (B) Normal self-grooming in Shank3Q321R/Q321R mice (4–5 months; male) in Laboras cages (a novel environment) with light (∼50 lux; 10 min). n = 9 mice (WT), 8 mice (Q321R), ns, not significant, Mann–Whitney U-test. (C) Normal self-grooming in Shank3Q321R/Q321R mice (2–3 months; male) in an open-field arena (a novel environment) with light (∼100 lux; 10 min). n = 13 mice (WT), 14 mice (Q321R), ns, not significant, Student’s t-test. [file Image_2.TIF]

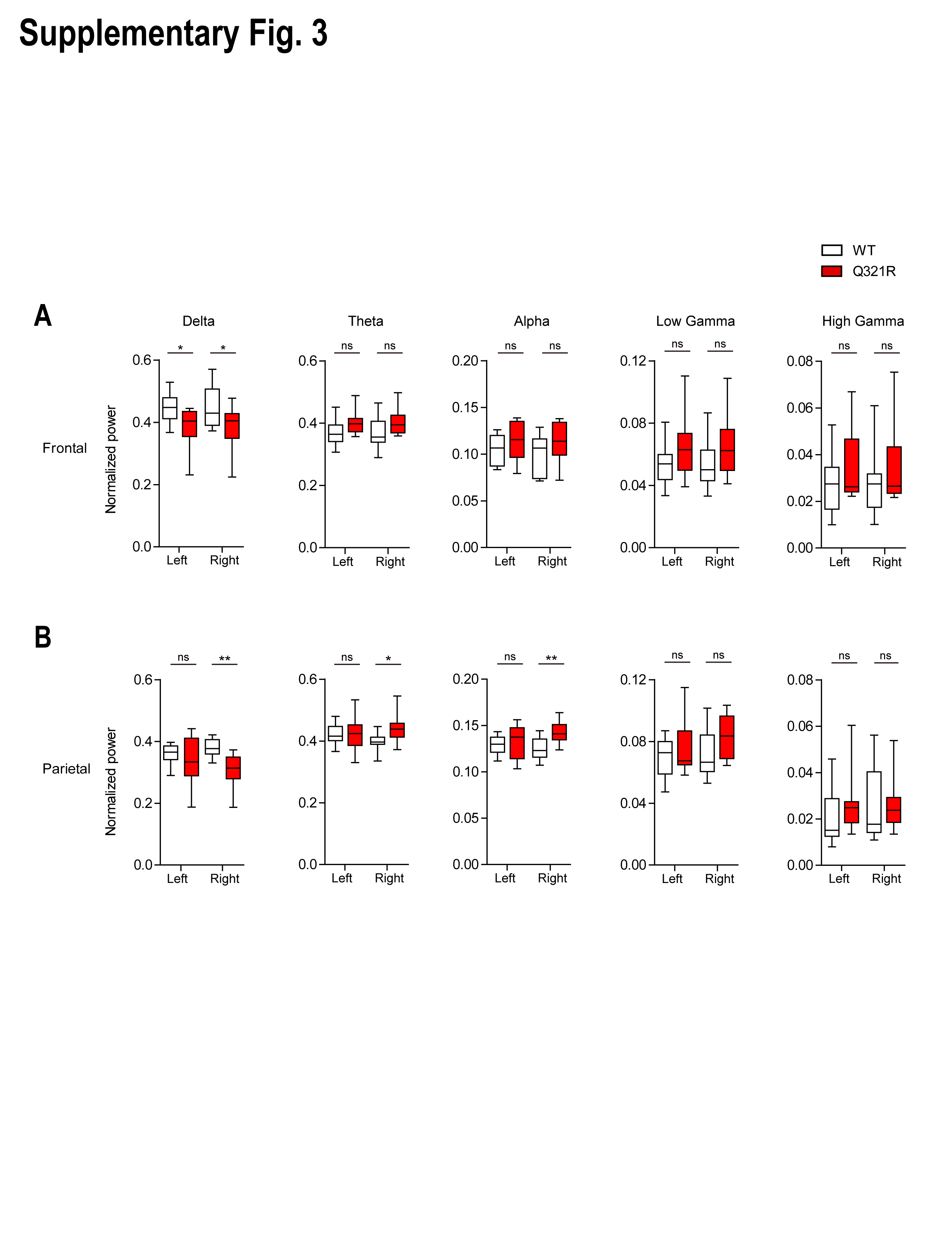

Supplement: FIGURE S3 — Abnormal EEG patterns in the left and right hemispheres in Shank3Q321R/Q321R mice. (A,B) Altered left and right hemispheric EEG powers in the frontal and parietal lobes of the Shank3Q321R/Q321R brain (3 months; male). Note that the frontal lobe displays decreased delta power in both left and right hemispheres, whereas the parietal lobe displays decreased delta power and increased theta and alpha powers only in the right hemisphere. n = 10 mice (WT) and 10 mice (Q321R) for frontal lobe and 9 mice (WT) and 10 mice (Q321R) for parietal lobe, ∗P < 0.05, ∗∗P < 0.01, ns, not significant, Student’s t-test, Mann–Whitney U-test, Welch’s t-test. [file Image_3.TIF]
